# MotifLab: a tools and data integration workbench for motif discovery and regulatory sequence analysis

**DOI:** 10.1186/1471-2105-14-9

**Published:** 2013-01-16

**Authors:** Kjetil Klepper, Finn Drabløs

**Affiliations:** 1 Department of Cancer Research and Molecular Medicine, Norwegian University of Science and Technology, Trondheim, Norway

## Abstract

**Background:**

Traditional methods for computational motif discovery often suffer from poor performance. In particular, methods that search for sequence matches to known binding motifs tend to predict many non-functional binding sites because they fail to take into consideration the biological state of the cell. In recent years, genome-wide studies have generated a lot of data that has the potential to improve our ability to identify functional motifs and binding sites, such as information about chromatin accessibility and epigenetic states in different cell types. However, it is not always trivial to make use of this data in combination with existing motif discovery tools, especially for researchers who are not skilled in bioinformatics programming.

**Results:**

Here we present MotifLab, a general workbench for analysing regulatory sequence regions and discovering transcription factor binding sites and *cis*-regulatory modules. MotifLab supports comprehensive motif discovery and analysis by allowing users to integrate several popular motif discovery tools as well as different kinds of additional information, including phylogenetic conservation, epigenetic marks, DNase hypersensitive sites, ChIP-Seq data, positional binding preferences of transcription factors, transcription factor interactions and gene expression. MotifLab offers several data-processing operations that can be used to create, manipulate and analyse data objects, and complete analysis workflows can be constructed and automatically executed within MotifLab, including graphical presentation of the results.

**Conclusions:**

We have developed MotifLab as a flexible workbench for motif analysis in a genomic context. The flexibility and effectiveness of this workbench has been demonstrated on selected test cases, in particular two previously published benchmark data sets for single motifs and modules, and a realistic example of genes responding to treatment with forskolin. MotifLab is freely available at http://www.motiflab.org.

## Background

Computational motif discovery for transcription factor binding sites is a challenging research problem that has been studied for many years, but we are still missing approaches that can ensure generally good performance. For transcription factors with known binding motifs, scanning sequences for matches to motif models can identify potential binding sites, but the performance is often strongly degraded by a high content of false positive predictions; predicted sites that do not correspond to actual transcription factor binding events [1]. *De novo* motif discovery, i.e. discovery of potentially novel motifs from a set of DNA sequences, can work well for input sequences with high motif content, like from ChIP-Seq experiments. However, it is often less successful on more general sequence sets, based for example on regulatory regions for co-regulated genes [2].

It is a commonly used approach to not rely on predictions of just a single method, but to run several motif discovery methods on the same dataset and compare the results. The motivation is, of course, that although one individual method might be mistaken in a single case, any motif predicted by several different methods is probably more likely to be correct. Tools such as Melina [3] and Tmod [4] provide users the opportunity of running and comparing results for several methods within a unified interface, and *ensemble methods*, like EMD [5] and MotifVoter [6], can take predictions from multiple methods as input and automatically derive a consensus. Still, the reason why motif discovery is so difficult in the first place is that binding motifs are often rather short and can vary substantially between binding sites. This makes them hard to discover with *de novo* motif discovery methods since the signal-to-noise ratio can be quite low when searching for motifs embedded in long background sequences. However, transcription factors seldom operate alone but work in concert with other transcription factors and co-factors in order to achieve the required regulatory control. Hence, groups of motifs for co-operating factors will often occur in close proximity to each other in the DNA sequence, and such “composite motifs”, or *cis*-regulatory modules (CRM), can provide a stronger signal than individual motifs. Several *module discovery* methods have therefore been proposed to search for such motif groups [7].

A fundamental limitation with the traditional motif and module discovery approaches is that they only rely on information in the DNA sequence itself, but the mere presence of a binding motif does not necessarily imply that it is a functional binding site. Other conditions, such as for instance chromatin accessibility, DNA-methylation or even the distance to the transcription start site, can also influence the ability of transcription factors to bind and exert their regulatory function. Many binding sites may also function in a cell- or tissue-dependent manner, and a site which is active in one cell-type might well be inactive in others.

Recent advances in high-throughput experimental methods and large-scale genome annotation efforts, such as the ENCODE project [8], have led to an avalanche of data which is now available to researchers. ChIP-Seq data, for instance, can provide evidence that a specific transcription factor has bound to a region (albeit perhaps by indirect binding), and information about DNase hypersensitivity and epigenetic marks can indicate which regions of the DNA are generally accessible and also give clues as to their regulatory roles in different cell-types.

Newer motif/module discovery methods, including for example Chromia [9], Centipede [10], ProbTF [11], CompleteMOTIFs [12], Combinatorial CRM decoder [13] and i-cisTarget [14], try to take advantage of such additional information in order to improve their predictions. Some of these tools rely on a fixed set of features which are utilized in a predefined manner. This makes them very convenient and easy to use, but it also means that they are unable to incorporate new data unless their original creators update the underlying databases. Other methods are more general and can work with arbitrary data, but require that the users themselves obtain all the relevant data for the sequences they want to analyse and also convert this data into a format the tool can handle. This might not always be a trivial task, and it can sometimes even require that the users are skilled in programming. Hence, the threshold for making use of additional data in the analysis can often be high.

In this paper we present a tool called MotifLab which is designed to be a general workbench for analysing regulatory sequences and predicting binding sites for individual transcription factors and modules of co-operating factors. The main purpose of MotifLab is to provide a flexible framework which allows users to easily incorporate different kinds of additional information into the motif discovery process. As a motif discovery workbench it has drawn inspiration from other related tools, primarily Toucan [15], but it also shares similarities with e.g. MochiView [16], SeqVISTA [17] and RSAT [18]. MotifLab is written in Java and will run locally as a stand-alone application.

## Implementation

### Software description

At its core, MotifLab functions as a repository of data objects that can be manipulated and analysed using a number of available *operations*. The results can be visualized and examined interactively within the system or be output to standard text based formats (FASTA, GFF etc.) for further processing by other programs. MotifLab is not backed up by a central dedicated database server, but data can be retrieved automatically from various internet resources, such as the UCSC Genome Browser [19] or DAS servers [20], or alternatively be imported from local files. New data objects can also be derived from already existing objects or created manually from scratch.

MotifLab distinguishes between several types of data for different purposes. One of the fundamental data types in MotifLab is the *sequence,* which represents a segment of a genome, such as the promoter region associated with a specific gene. Users can create new sequence objects by specifying their chromosomal coordinates or by providing MotifLab with a list of gene identifiers and selecting a region to analyse around the genes’ transcription start or end sites. The sequence objects merely function as references to genomic locations, and besides the coordinates, genome build and strand orientation of the sequences, they hold little additional information by themselves. However, sequences can be further annotated with *feature datasets*, which come in three different types: *DNA sequence datasets*, *numeric datasets* and *region datasets*. *DNA sequence datasets* contain a single base letter for each position within a sequence. Usually, they just hold the original DNA sequences for the genomic segments being investigated, but it is fully possible to have multiple DNA sequence datasets associated with the same sequences. These additional datasets can then contain masked versions of the original DNA sequence or randomly scrambled sequences to be used for statistical comparisons. *Numeric datasets*, on the other hand, have a numeric value for each position in the sequences, and this data type can represent information such as phylogenetic conservation level, DNA stacking energy, melting temperature or basically any other signal that can vary in intensity along the sequence. The final feature data type, *region datasets*, associates each sequence with a set of regions. A *region* here refers to a subsegment of a sequence which has distinct properties that sets it apart from the rest of the sequence. Regions can represent features such as genes, CpG-islands, repeat regions or transcription factor binding sites. Different regions within the same sequence may overlap each other, and regions can also be assigned values for various attributes, including a type designation, score value and strand orientation.

**Figure 1:**
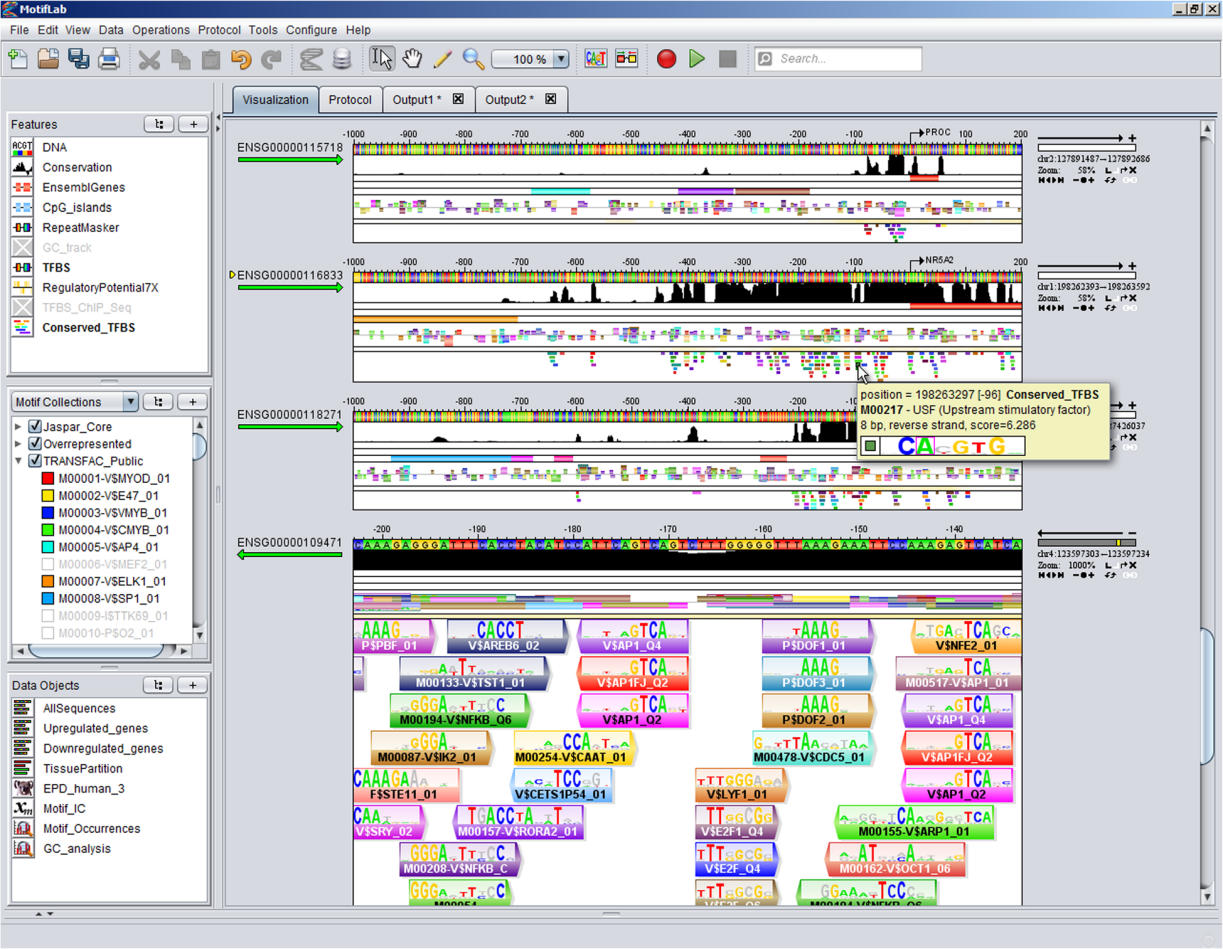
**MotifLab’s graphical user interface.** The screenshot shows MotifLab’s graphical user interface with three data panels to the left and with the sequence browser to the right taking up most of the screen space. The top data panel contains the feature datasets in the order they are visualized as tracks in the sequence browser, the middle data panel contains the motifs and modules, and the bottom panel contains miscellaneous data objects that to not belong in the first two panels. Features and motifs that are greyed out in the data panels are hidden from view in the sequence browser. The bottommost sequence shows a motif track in “close-up mode” which is activated at zoom-levels above 1000%. The binding sites are shown with superimposed “match logos” where the base matching the DNA sequence in that position is shown in colour and the other three bases are greyed out.

MotifLab’s graphical user interface offers a sophisticated sequence browser with powerful capabilities for visualizing sequences and associated feature data tracks, as shown in Figure 1. All the sequences are displayed simultaneously beneath each other in the same window so that features for different sequences can be compared visually. The browser is highly interactive and customizable, and it supports fast zooming to any scale and panning to show different parts of a sequence. The appearance of each track, including its colour, size and orientation, can be easily modified, and the order of the tracks and sequences can be rearranged or sorted according to different criteria. Individual sequences, tracks and even individual regions within region datasets can also be hidden from view to display only what the user wants to focus on at any time.

Besides sequences, another fundamental data type is the *motif*, which is used to model binding motifs for transcription factors. The binding motifs themselves are typically represented as either position weight matrices or IUPAC consensus sequences, but motif objects can be annotated with a lot of additional information as well, such as the names of different transcription factors that bind to the motif, names of organisms and tissues these factors are expressed in, references to other motifs representing known interaction partners for these factors and references to alternative models for the same motif. New motif objects are automatically added when performing motif discovery, but they can also be created manually by entering a matrix, IUPAC consensus or a set of aligned binding sequences. MotifLab includes several predefined motif models from databases such as TRANSFAC [21], JASPAR [22] and ScerTF [23].

It is often useful to be able to refer to subsets of sequences and motifs, for instance to divide a set of sequences into groups according to gene expression or to limit the search for binding motifs to transcription factors that are actually present in the cell-types being investigated. In MotifLab this can be accomplished with the help of *collection* objects. Users can create new collections by selecting data objects from a table or by supplying a list of objects to include. Collections can also be based on various statistics. For example, it is possible to create a sequence collection containing sequences with less than 40% GC-content or a motif collection with motifs that appear in at least 80% of the sequences. Somewhat related to *collections* are *partitions* which allow all data objects of a specific type to be divided into non-overlapping clusters. The *numeric map* data type associates each sequence or motif with an individual numeric value. Numeric maps can be used to hold data such as gene expression values for sequences or expected occurrence frequencies for motifs. General *text variable*s, on the other hand, can hold any kind of structured or unstructured text which will be interpreted depending on the context.

### Operations and protocol scripts

MotifLab provides more than 40 data-processing operations to create, transform, combine, analyse and output data objects, including special operations to perform motif and module discovery. Some of these operations, like “output” and “copy”, can be applied to any object, while others may be specific to a single type of data. The “mask” operation, for instance, can replace parts of a DNA sequence with other letters, such as X or N, or it can even replace the whole sequence with random bases sampled from a background distribution to create an entirely new artificial sequence. Numeric data objects can be transformed with arithmetic operations or other mathematical functions such as logarithms, range normalizations etc., and sliding windows can be applied to numeric features to smooth the data or to detect peaks, valleys and edges within the track. Other operations can change the size of regions by extending them in either direction or merge regions that overlap with each other. One of the simplest operations, but also one of the most useful, is the “filter” operation, since it can be employed to remove selected regions from a dataset, particularly binding sites that are suspected to represent false predictions.

Operations that target feature datasets can be limited to selected parts of sequences by specifying *conditions* that are evaluated for each individual base position or region in the dataset. These conditions can be based on the contents of the target track itself or involve information compared across several tracks. For example, a simple way to perform phylogenetic footprinting without having explicit access to orthologous sequences would be to first predict a set of binding sites in the normal way and then filter out those predictions where the average value of a conservation track within the binding sites is less than some threshold. Likewise, the process of “repeat masking”, which is often performed prior to motif discovery, can easily be accomplished by limiting the “mask” operation to bases that lie within regions in a track containing known repeats. Conditions offer an easy way of integrating information from several features, and they can be made arbitrary complex by combining multiple individual conditions with Boolean operators.

Analysis of regulatory sequences usually involves multiple steps and requires several operations to obtain and pre-process data, discover motifs and binding sites and post-process and analyse the results. To keep track of what is being done, MotifLab provides functionality that allows users to automatically record every step they perform in a *protocol*. The protocol is written in a structured format and includes information about which operations have been executed, as well as details about their parameters, conditions and constraints. The protocol can thus serve as a form of documentation of the analysis process, but more importantly, it also makes MotifLab able to automatically apply the same workflow to other datasets as well, or to restore a previous session. Protocols can alternatively be written and edited manually, either in external text editor programs or in MotifLab’s own internal protocol editor. By supplying a protocol script describing the full analysis workflow, it is possible to run MotifLab in “batch mode” from a command line without starting up the graphical user interface. This also allows MotifLab to be incorporated as a component in larger analysis pipelines.

MotifLab’s graphical user interface promotes interactive data exploration, and multi-level undo/redo-functionality provides users the opportunity to experiment with various operations and try out different parameter settings for these without having to worry about making irreversible changes to the data. Unlike some other workbench systems, MotifLab does not maintain an explicit history record which keeps track of all changes made to data and provides access to earlier states. However, when data is updated through the use of operations, the results can always be stored in a new data object under a different name rather than replacing the original object. This way, the original data can be kept intact and used for other purposes as well. It is also possible to save the entire state of MotifLab to a single “session file” to continue working on an analysis at a later time.

### Motif discovery

Discovering motifs and searching for transcription factor binding sites within sequences are some of the primary functions of MotifLab. However, MotifLab is not actually capable of performing motif discovery by itself but relies on external programs installed on the user’s computer to accomplish such tasks. This makes MotifLab flexible with respect to local software preferences or novel tools. In order for MotifLab to communicate with external programs, they must conform to standard data formats for input and output and their interfaces must be described in XML-based configuration files. MotifLab already supports several popular motif discovery tools, including AlignACE [24], BioProspector [25], MDscan [26], MEME [27], MotifSampler [28] and Weeder [29], and more tools will continuously be added (visit the MotifLab web site for a complete and updated list). Many of the supported programs have also been gathered in a central repository so they can be downloaded and installed from within MotifLab.

MotifLab has separate operations for performing *motif scanning,* where external programs are provided with a collection of predefined motifs and should return a region dataset containing predicted binding sites for these motifs, and *de novo motif discovery*, where the programs should discover both the binding sites and the motifs themselves. In addition, a third operation offers support for *ensemble methods* which can take predictions from other methods as input and combine these into potentially more reliable predictions.

Tracks with predicted binding sites are called *motif tracks,* and they have a special status in MotifLab because of the connection between the binding site regions and the motif objects associated with these sites. This enables the sequence browser to visualize binding sites with motif logos superimposed on the regions (as can be seen in the bottom sequence in Figure 1), and clicking on a binding site will bring up additional information about the motif.

### Using positional priors to guide motif discovery

Motif discovery is a challenging problem since it involves searching for short and often degenerate patterns embedded in potentially long sequences. However, some parts of the sequences are more likely to contain functional binding sites than others, such as regions where the chromatin has an open conformation or sites that have been conserved throughout evolution. Some motif discovery programs allow users to limit the search space by masking out parts of sequences and thereby excluding them completely from further consideration. However, this approach might be considered too strict, since the excluded regions could, in fact, contain functional binding sites that will inevitably be destroyed by the masking procedure. A more flexible alternative is to construct a *positional priors* track wherein each sequence position is assigned a score or probability value reflecting a prior belief that the position could be part of a binding site. Such a track can be used to guide motif discovery programs by biasing the search towards regions with higher probability of containing true sites. Many types of information can be represented using positional priors, for instance phylogenetic conservation [30], nucleosome occupancy [31], properties of the DNA-helix [32] and epigenetic marks [33], and information from many different sources can be combined into a single priors track [34]. Positional priors are currently only supported directly by a few motif discovery and scanning programs, including PRIORITY [35], MEME [36], FIMO [37], ChIPMunk [38] and GRISOTTO [39], but they can also be used indirectly in combination with other programs, for instance by employing positional priors to filter out likely false predictions in a post-processing step.

Although tracks related to e.g. conservation, DNase hypersensitivity and ChIP-Seq experiments do not actually contain probability values in a strict statistical sense, such tracks can often be used directly as positional priors (or after minimal processing) since higher values in these tracks correlate well with occurrences of functional binding sites. For other types of features the relationship might not be so direct, and more advanced processing will be required to generate positional priors based on such features. MotifLab is an extension of an earlier program called PriorsEditor [40], which was developed specifically for creating and using positional priors tracks for motif discovery. Many of the operations provided by MotifLab are therefore related to transforming and combining features to facilitate manual construction of positional priors tracks, for instance to make weighted combinations of several tracks. Creating positional priors tracks manually can be beneficial if you want to utilize specific biological knowledge or want to set up a track with clearly defined focus. For example, if you have a set of known binding sites for a single transcription factor and want to look for potential interaction partners for this factor, you can create a track which focuses the search to the vicinity of these sites, possibly adjusting the track further, for instance by assigning increased weight to conserved regions.

Compared to PriorsEditor, MotifLab offers several new functions to work with positional priors, including an operation to convert a regular priors track into a *discriminative prior* (as described in [31]) and analyses to evaluate the potential merit of priors tracks. The most important new addition, however, is the introduction of “Priors Generators” that can be used to generate positional priors automatically based on information from various features. A Priors Generator is basically just a machine learning classifier that can be trained to predict whether or not a position in a sequence would be expected to lie within a transcription factor binding site depending on the values of relevant features at that position. MotifLab provides a simple “wizard” to guide users through the steps required to configure a new Priors Generator, such as selecting the target and input features, setting up a training dataset and finally training the classifier and saving the result. Once a Priors Generator has been created, it can be used to generate positional priors for any sequence as long as the required input features are available. Although Priors Generators were introduced primarily for the prediction of transcription factor binding sites, they can just as well be trained to predict other region-based features in the same manner, provided that a reasonable correlation between the target feature and the input features can be expected.

### Module discovery

Co-occurrence of motifs in modules represents a higher level of *cis*-regulatory organization that can be exploited to improve motif prediction, as binding sites for interacting factors which appear in close proximity to each other are less likely to represent spurious motif occurrences. MotifLab allows motifs to be annotated with information about known interaction partners, and one way to utilize this information is simply to filter out predicted binding sites that do not have sites for potential partners within some given distance.

Regulatory modules can also be modelled explicitly in MotifLab with their own data type analogous to single motifs. A *module* is made up of multiple constituent motifs along with optional constraints on their order, their orientations relative to each other and the distances between them. Because public motif databases often contain several alternative motif models for the same transcription factors, MotifLab permits each constituent motif in a module to be represented by collections of motifs in order to achieve greater sensitivity when performing module scanning.

As for single motif discovery, MotifLab provides separate operations to scan sequences for matches to predefined modules and to search sets of sequences to identify groups of motifs that might represent novel modules. Again, both of these operations rely on external module discovery programs to do the actual work.

### Statistical analyses

The *analyze* operation is a versatile operation that can be employed to perform a number of different statistical analyses ranging from simple data comparisons to more elaborate analyses like motif overrepresentation studies. It will often be used to produce the final reports for an analysis session, but it is also useful for providing rapid answers to simple questions that might arise when working with datasets, such as “what is the GC-content of these DNA sequences”, “do these two collections share a significant overlap”, “is property X correlated with property Y” or “is the value of this numeric track higher within some regions than outside”.

The results from the analyses can be output either as HTML-documents, with nicely formatted tables and images, or in a “raw text” format suitable for parsing by other programs. Individual results can also be extracted from analysis objects and turned into other types of objects for use elsewhere. For example, if you have performed an analysis to determine the number of times each motif occurs in a sequence set (“count motif occurrences”), you can extract these counts as a *numeric map*, or you can make a *motif collection* containing the motifs that were significantly overrepresented in the sequences and use this in another analysis.

Some analyses, like the previously mentioned “count motif occurrences”, will generate individual results for each motif, module or sequence, and these results are presented in interactive tables that are linked to the corresponding data items. This makes it possible to e.g. highlight entries in the tables that are members of different collection objects, or to highlight corresponding elements in the sequence browser based on selections made in the tables. For example, when examining the results from a motif overrepresentation analysis, users can select the top most significant motifs from the table and then choose “Show only these motifs” from a context menu to visualize only the binding sites for these motifs in the sequence browser. The tables are therefore not merely static presentations of the results, but can be used as a starting point for further exploration of the data. If the tables contain motifs, the motif logos will always be included in a separate column. This is very useful, since rather than just listing numerous motif identifiers or names of transcription factors the user may or may not be familiar with, the logos enable users to immediately identify properties of the corresponding motifs and see similarities between them.

Results from multiple analyses can be collated into “meta-analyses” by extracting selected columns from individual analyses and combining them into larger tables. Information from different types of analyses can be combined in this way to produce more comprehensive reports, or results from the same analysis run multiple times with different parameter settings can be juxtaposed to assess the impact of varying these parameters.

### Interactive tools

In addition to the data manipulation and analysis capabilities provided by operations, MotifLab also includes a few tools aimed at interactive exploration of data. Unlike operations, these tools cannot be controlled by protocol scripts, and they are only available through the graphical user interface. Many of the tools are intended to aid visual inspection of motif tracks, for instance by highlighting binding sites with selected properties in the sequence browser.

**Figure 2:**
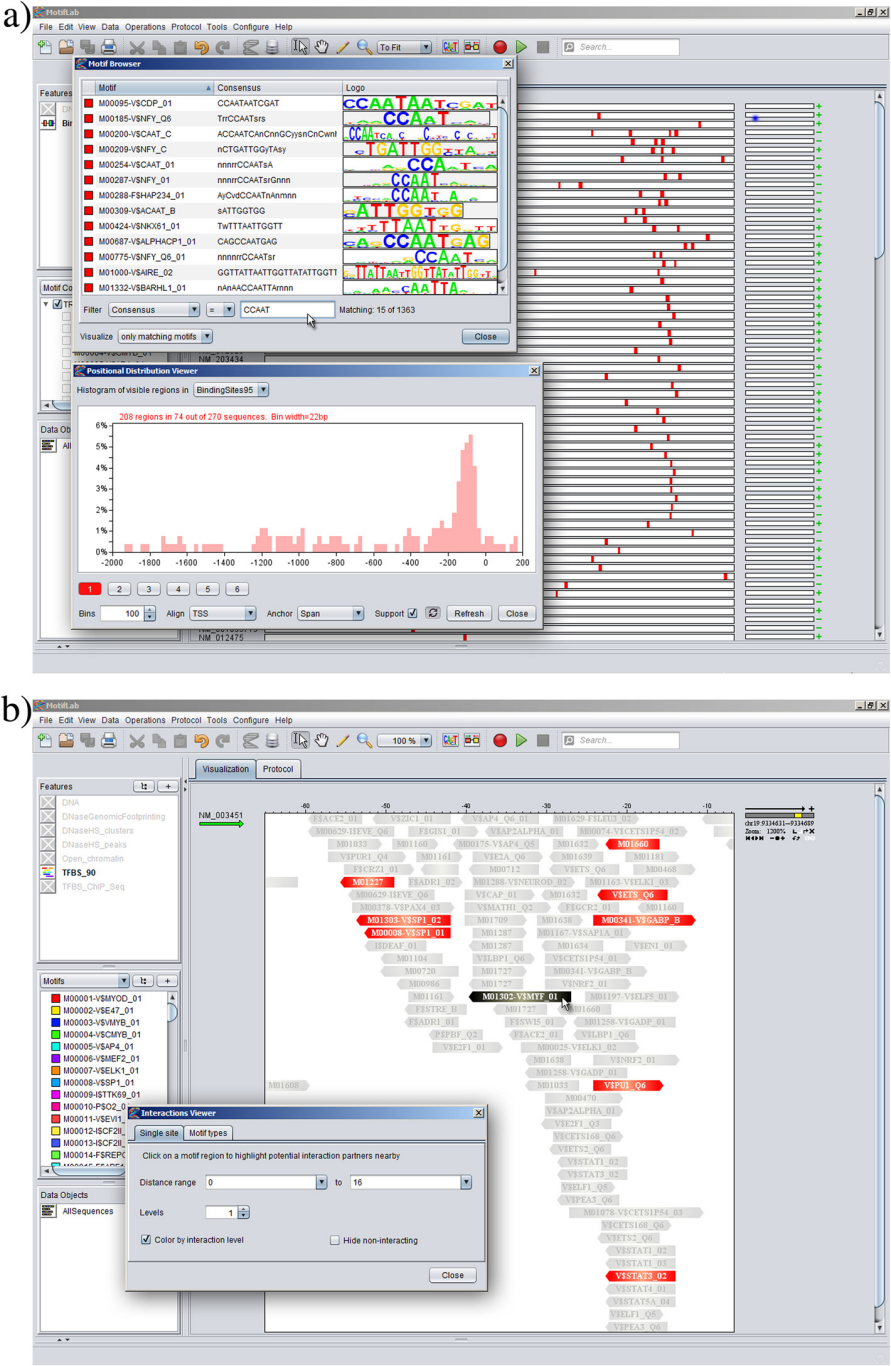
**Examples of interactive tools. a**) The **Motif Browser** tool (top dialog box) has here been used to search for TRANSFAC motifs containing the consensus sequence “CCAAT” (both orientations). The corresponding binding site predictions for the 15 motifs matching this criterion are shown as red boxes in the sequence browser partly visible in the background. The **Positional Distribution Viewer** tool (bottom dialog box) shows a histogram of the locations of these binding sites, and the prominent peak indicates that the majority of the sites are located within 200 bp upstream of the TSS. **b**) The **Interactions Viewer** tool. A part of a motif track is shown in close-up mode at 1200% scale (binding sites are displayed here without motif logos). The black binding site in the middle is the target site selected by the user and the red sites on either side have been highlighted by MotifLab as binding sites for transcription factors that are known to interact with the target factor(s) from other locations. All other binding sites are greyed out.

The **Motif Browser** and **Module Browser** are two convenient tools for managing your motif and module libraries. These browsers will show an overview of all motifs or modules currently known to the system. The entries are displayed in a table with three columns containing the name of the motif/module, a graphical logo representation, and a third property that can be chosen by the user (see Figure 2a). A filter box enables users to search for entries with specific properties, for instance motifs associated with a given transcription factor, motifs for factors expressed in specific organisms or tissues, motifs containing a given consensus sequence, or modules containing a specific constituent motif. The search filter can also be coupled to the sequence browser so that only binding sites for motifs or modules matching the selected filtering criteria will be shown in the tracks.

The **Motif Score Filter** tool is basically just a slider bar which is used to dynamically adjust a cut-off threshold. Any binding site region whose score-property falls below the selected threshold will be hidden from view in the sequence browser. This tool can thus be used to highlight sites with increasingly higher scores. Besides the standard score-property, other values associated with binding sites can be used for filtering as well, for instance the average score of a numeric data track within the binding site.

As previously mentioned, MotifLab allows motifs to be annotated with known interaction partners, and this information can be utilized by the **Interactions Viewer** to visualize potential interaction networks directly within motif tracks. When a user clicks on a binding site region, any binding sites within a chosen distance that are associated with known interaction partners of the target motif will be highlighted (Figure 2b). The network can also be expanded to show several levels of interactions in different colours. This tool is especially useful if you already have a verified binding site that can be used as a starting point to implicate additional predictions that might be likely to represent functional binding sites.

Finally, the **Positional Distribution Viewer** will draw a histogram based on the locations of all currently visible regions in a selected track across all sequences (Figure 2a, bottom). The histogram will be dynamically updated in response to events that change the visibility of regions, making it very useful in conjunction with other tools such as the Motif Browser or Motif Score Filter.

## Results

This section presents three examples of practical applications using MotifLab, which also illustrate some benefits of incorporating additional information when analysing regulatory sequences. Complete protocol scripts for these examples are available from the MotifLab web site.

### Example 1: Improving motif discovery with automatically generated positional priors

We have previously published a suite of benchmark datasets for single motif discovery (based on binding sites annotated in TRANSFAC) where we made sure that it would be at least theoretically possible to discriminate the target motifs from the background sequence. Nevertheless, when we tested the performance of the motif discovery program MEME on these benchmark sets, the results were not particularly encouraging [41]. In this example we use an updated version of the datasets (see Additional file 1) to demonstrate how information about various sequence-related features can be integrated into a positional priors track and used to guide MEME towards the target motifs. The features chosen as a basis for the priors track were: conservation, conserved peaks, DNase hypersensitive sites, general regions bound by transcription factors according to ChIP-Seq data, CpG-islands, gene regions, coding regions, repeat regions and regions with histone marks H3K4me1 and H3K4me3. Since not all organisms are annotated with these features at the present time, we restricted the benchmark datasets to only consist of sequences from human and mouse genomes. The updated benchmark suite comprised 22 datasets, each containing binding motifs for a particular transcription factor and consisting of at least five sequences. We used a cross-validation approach where a Priors Generator based on a neural network classifier was trained on 21 of the 22 datasets and then used to generate a positional priors track for the dataset that was held out. The priors tracks were provided as input to MEME along with the DNA sequences, and MEME was instructed to identify a single motif with size between 8 and 16 bp in each dataset. For comparison we also ran MEME with a uniform priors track (effectively the same as using no priors) and a priors track based solely on conservation.

**Figure 3:**
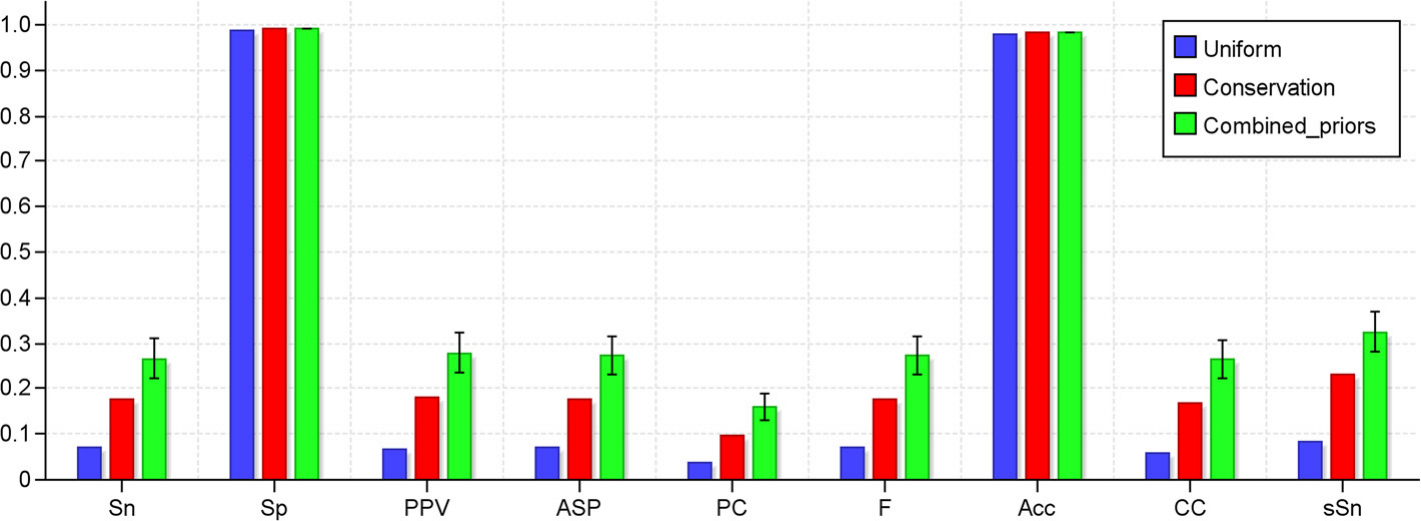
**Results from example 1 – Single motif discovery benchmark.** The figure shows the performance of MEME on the single motif discovery benchmark when guided respectively by a uniform positional priors track, a priors track based only on conservation, and a combined priors track made by automatically integrating information from several features with the use of a Priors Generator. The statistics were calculated by combining all sequences from the 22 datasets into one large dataset and measuring the overlap between the predicted binding sites and the target sites. The first eight statistics are *nucleotide*-level statistics whereas the last statistic is the *site*-level sensitivity (number of predicted sites overlapping with at least 25% of a target site). Due to the stochastic nature of the algorithm used to train the Priors Generator, the combined priors track could vary slightly depending on the training. We therefore trained 20 different Priors Generators and ran MEME with priors tracks generated by each of them. The bars show the average scores with standard deviations over the 20 runs.

**Figure 4:**
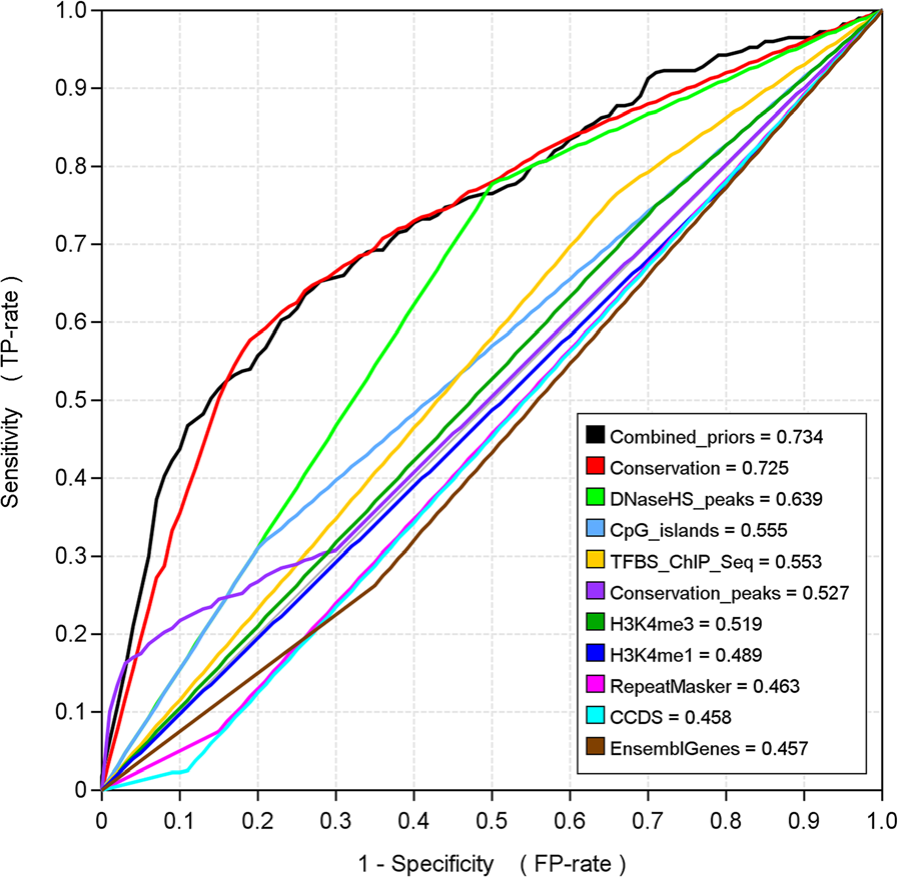
**Predictive capabilities of individual features.** These ROC-curves illustrate the ability of both the auto-generated combined priors and the 10 individual features the combined priors were based on to discriminate between sequence positions that are part of binding sites or part of the background sequence. The numbers in the legend box are the *area under the curve* (AUC) values for each feature.

The results, combined over all datasets, are shown in Figure 3. Detailed results for individual datasets are provided in Additional file 1. As can be seen from the bar chart, the performance of MEME when not relying on any additional information was rather low, with an average CC score of 0.06. However, the performance increased about 3- to 4-fold for most metrics when the automatically generated positional priors were used to guide the search. Many of the target binding sites in the benchmark were located in conserved regions, and conservation was the most informative single feature with respect to binding site prediction. Conservation also contributed most to the specificity of the combined priors, while the other features primarily helped to elevate the basal prior probability slightly within some broader parts of the sequences. Figure 4 illustrates the ability of the individual features to discriminate between binding sites and the background sequence.

Even when positional priors were used, the results were far from perfect. There are several reasons for this: 1) for about half of the datasets MEME failed to predict the correct target motif as its top candidate, 2) in some datasets where MEME did identify the correct motif, alternative binding sites for the motif were selected instead of the annotated targets in a few sequences, and 3) even if MEME predicted the basic motif and binding sites correctly it did not always predict the correct size of the motif, which could have significant impact on the nucleotide-level statistics. A closer look at the predicted sites and motifs revealed that MEME found the target motif (or a resembling one) in 3 out of the 22 datasets with the uniform priors. This number increased to 8 when conservation was used to guide the search, and with the auto-generated priors MEME found the target motif in about 9 to 11 datasets (depending on the particular Priors Generator used).

### Example 2: Module discovery

In a second benchmark study we evaluated the performance of eight published module discovery methods on a novel benchmark suite [7]. The suite consisted of 10 datasets with pairs of motifs appearing together in multiple sequences and two additional datasets with larger heterogeneous modules involved in regulation in liver and muscle tissue respectively. Most of the methods we tested relied on a first step to scan sequences with a provided motif collection to find a set of candidate binding sites, and then they proceeded to search through these candidates in order to identify potential modules. The results showed, not surprisingly, that the task of identifying the target modules became harder as more candidate motifs were considered. In this example we demonstrate how the performance of a module discovery tool can be improved by utilizing additional information to reduce the number of candidate sites in a pre-processing step.

To generate the candidate datasets we first scanned the benchmark sequences with 1363 motifs from TRANSFAC PRO using a rather sensitive threshold (80% match) to ensure that all the target binding sites were recovered. Then we filtered the predicted sites according to various criteria to produce different candidate sets. As filtering criteria we used increasing levels of average conservation within the sites (more than 0%, 10%, 30% and 60%) or required that each site should be located nearby a site for a known interaction partner (within 10 or 20 bp). For the “liver” and “muscle” datasets we also filtered sites for transcription factors that were not known to be expressed in the respective tissues. In addition we tried several combinations of these criteria. The remaining binding site predictions for each dataset were provided as input to the module discovery tool ModuleSearcher [42].

**Figure 5:**
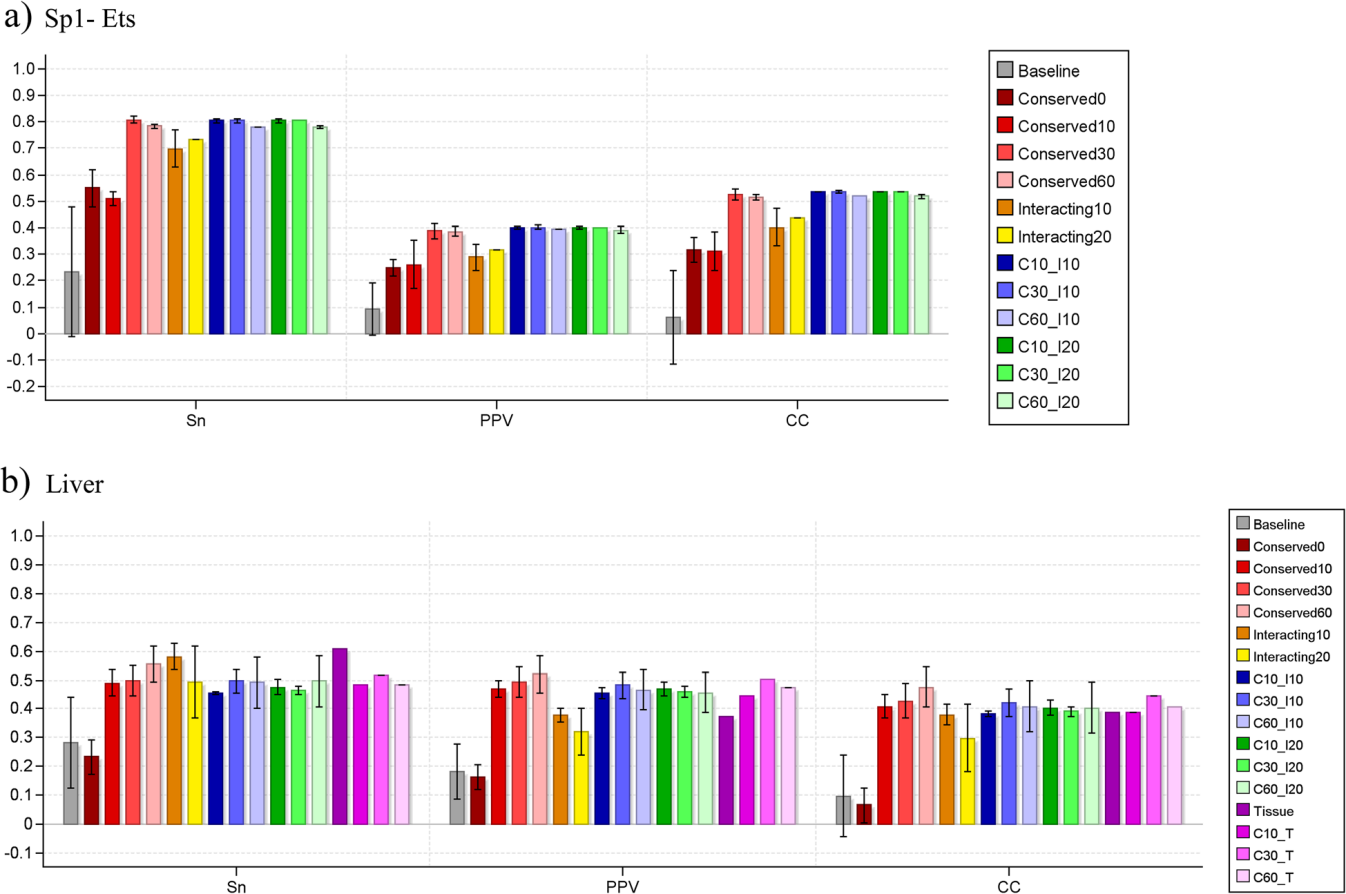
**Results from example 2 – Module discovery benchmark.** This figure shows the nucleotide-level performance of ModuleSearcher on two of the datasets from the module discovery benchmark. Since ModuleSearcher is based on a non-deterministic algorithm, we ran it 10 times on each dataset. The bars show the average scores with standard deviations. The “baseline” scores reflect the performance when no pre-processing was performed to filter candidate binding sites, and the other scores are for different filtering criteria and combinations thereof. “C10_I10” means that the sites were filtered according to both the “Conservation10” and “Interacting10” criteria etc. **a**) The “Sp1-Ets” dataset was one of the hardest in the original benchmark, but filtering sites based on either conservation or potential interacting sites nearby significantly improves the performance of ModuleSearcher on this dataset. **b**) For the “liver” dataset we also filtered binding sites for motifs that were not known to be expressed in liver (“Tissue”) and combined this criterion with different requirements on conservation level (“C10_T” etc.).

Results for two of the datasets are shown in Figure 5, and the remaining results are included in Additional file 1. For all 12 datasets there was some form of additional information that would lead to better performance when used to filter candidate sites. However, in some cases, there were filtering criteria that would actually result in lower performance. This was especially true for the datasets “CEBP-NFkB” and “IRF-NFkB” (see Additional file 1: Figures S2d and S2g). These two datasets were the easiest in the original benchmark, and ModuleSearcher did a good job of discovering the target modules even without filtering the candidate sites. However, since only about half of the binding sites comprising these modules were conserved, using a strict conservation criterion made it impossible to correctly discover the target modules.

As an additional control we also tried to filter the candidate datasets completely at random to verify that any increase in performance was not simply due to a general reduction in the number of candidate sites. Filtering sites at random would in fact lead to better results in many cases, most notably for datasets where the baseline performance was poor. This is perhaps not so surprising, since the vast majority of the candidate sites would be considered to be false positives anyway according to the benchmark datasets. However, the increase in performance was usually not as great as when more sensible filtering criteria were employed.

### Example 3: Identifying TFs regulating genes after forskolin treatment

Forskolin is a diterpene which is known to raise the level of cAMP (a second messenger) within cells [43], and this will in turn trigger many different responses, including activation of various transcription factors. HEK293 cells were treated with forskolin and the effect on gene expression was measured at different time points using microarray technology. Genes that were significantly differentially expressed compared to untreated cells were identified and sorted according to their peak time point. Of the 860 genes in total whose transcript levels were changed by the forskolin treatment, 270 had a peak differential expression after 2 hours (108 upregulated and 162 downregulated). We obtained promoter sequences for these 270 genes spanning 2000 bp upstream to 200 bp downstream of the transcription start site and performed motif scanning with 931 vertebrate motifs from TRANSFAC PRO.

The standard procedure for identifying transcription factors that might be involved in regulating a set of genes is to identify motifs that are significantly overrepresented in the dataset relative to a realistic background frequency. To estimate an expected frequency of each motif, we used a 3rd-order background model based on human promoter sequences to create a set of artificial control sequences and performed motif scanning in those sequences using the same parameter settings as before. We then derived the frequencies of the motifs from these control sequences and stored the results in a *numeric map*. Finally, we counted the number of times each motif occurred in the target dataset and used the expected motif frequencies to calculate p-values for overrepresentation with a binomial test. 113 motifs were found to be overrepresented at a significance threshold of 0.05 (Bonferroni corrected to 5.37 × 10 ^–5^ by dividing with the number of motifs considered). Many of the motifs with lowest p-values were GC-rich, which might stem from the fact that the sequences in the target dataset had a slightly higher GC-content than the control sequences used for comparison. The transcription factor CREB, which is a well-known cAMP-responsive factor but does not have a particularly GC-rich motif, was only ranked as number 57 according to p-value.

An alternative to overrepresentation is to look for motifs whose sites share similar properties across several sequences, for instance motifs that tend to appear at the same distance from the transcription start site, or motifs that are consistently conserved in many sequences. We therefore ran two additional analyses where we first calculated the average conservation level for each motif across all its binding sites and then analysed the positional distribution of the sites, using *kurtosis* as a simple measure of clustering.

Not surprisingly, the motifs that scored highest on average conservation were those that only occurred once or twice and their binding sites just happened to lie within conserved regions. These motifs are not interesting for the dataset as a whole, however, since they are at best involved in regulating only a few genes. The most interesting motifs would be those that score high on conservation and kurtosis but still occur often enough to have a significant overrepresentation p-value, so we combined these three properties into a single measure using rank sum.

According to this combined measure, CREB (along with the related factor ATF which binds to the same motif) was ranked on top, followed by the ubiquitous factor Sp1 which binds to the GC-box. Another significant transcription factor found was NF-Y which binds to the CCAAT-box. This motif scored particularly high on kurtosis, and it is known that functional binding sites for NF-Y tend to be located between 60 to 100 bp upstream of the TSS [44]. NF-Y is also known to cooperate with Sp1 to regulate some genes in response to cAMP [45,46]. The two factors NRF-1 and NRF-2 (nuclear respiratory factors) bind to different motifs, but both are ranked high and both have previously been implicated together with CREB in responses to raised levels of cAMP [47]. Interestingly, many of the sites for these two factors coincided with narrow peaks in the conservation track whose size matched exactly the width of the motifs. The fact that these sites were conserved while the flanking sequence around the sites was not is a strong indication that the sites might be functional.

**Figure 6:**
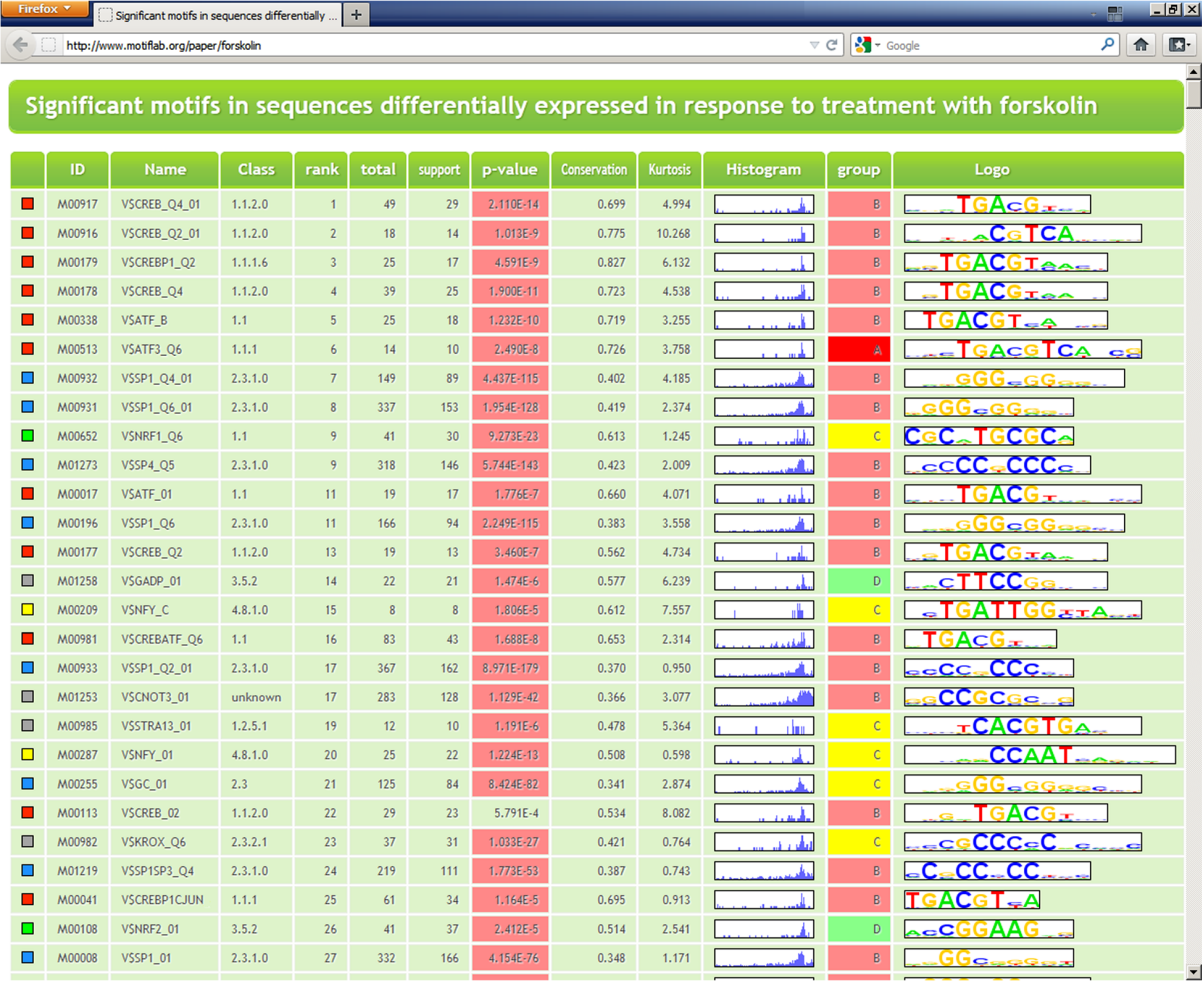
**Results from example 3 – Genes responding to forskolin treatment.** Results from the forskolin-analysis output in HTML format. The table is a combination of results from four different analyses performed in MotifLab. The “total”, “support” and “p-value” columns are from an analysis that counts the number of times each motif occurs in the sequences and estimates the significance of overrepresentation (significant p-values are highlighted in red). The “conservation” column is the average score taken from an analysis that compares the binding sites for each motif to a selected numeric feature (here conservation). The “kurtosis” and “histogram” columns are from an analysis of the positional distribution of the binding sites for each motif. The “group” column is from an analysis that compares the number of binding sites for each motif within sequences from two different groups to see if some motifs are overrepresented in one group compared to the other. Here we compared the group of upregulated genes to the downregulated genes. Motifs in the “A” and “B” groups (in red) were significantly overrepresented in the upregulated sequences whereas motifs in the “D” group (in green) were overrepresented in the downregulated sequences. Motifs in the “C” group (yellow) occurred at approximately the same rates in both groups. The table is sorted according to the combined ranks of p-values (ascending), conservation (descending) and kurtosis (descending). Note that almost all top ranking motifs are preferentially located within a narrow region upstream of the TSS, as indicated by the sharp peaks in the histograms around this position. Motif types are colour coded in the left-most column (CREB/ATF motifs with boxes in red, SP1 in blue, NF-Y in yellow, nuclear respiratory factors in green, others in grey).

Figure 6 shows the top ranking motifs from this analysis. The full table is available on-line at the MotifLab web site.

## Discussion

The examples given above, as well as previous publications by other groups, have shown that making use of additional information might boost the performance of motif and module discovery methods and help steer them towards regulatory elements that are more likely to be functional in a given context. However, relying on the “wrong” data, or even using data in the wrong context, can sometimes also have adverse effects. For example, filtering predicted sites based on phylogenetic conservation can lead to a higher proportion of true sites among the remaining predictions, but this will inevitably also remove any functional sites that are species-specific, and therefore not conserved. Even “gold standard” data, like DNase hypersensitive sites, should be used with some caution, especially when applied across different cell-types and conditions. To help users decide on which types of data might be useful to consider, MotifLab includes several analyses to evaluate the merit of different types of information and to benchmark the performance of motif and module discovery methods. In fact, all the performance evaluations in the previous examples were performed within MotifLab, and the bar chart figures and ROC-curves included in this paper were produced directly from the analyses using the “output” operation.

Although many recent motif discovery tools can make use of additional data, they are often limited in what kind of data they can use and what they do with it, typically using information about known repeats to mask sequences or conservation to filter predicted binding sites. MotifLab allows users to incorporate many different types of data and use it in any way they like. No kind of information is treated as special compared to others by MotifLab, and information is represented with a few general data types. This means that it should be easy to also incorporate new kinds of data that might be available in the future.

The ability of MotifLab to process data in arbitrary ways using operations also sets this tool apart from most other motif discovery workbenches. The program has been designed so that users with some background in the field of regulatory sequence analysis should be able to rapidly learn how to perform standard tasks such as obtaining promoter sequences, annotating them with feature data and performing motif discovery or scanning. But it should also be relatively easy to perform more sophisticated pre- and post-processing tasks which would otherwise often require writing custom scripts. For the use case examples described in this paper, all the data processing steps involved in the analyses were performed within MotifLab itself.

MotifLab keeps all data objects in memory at all times rather than relying on external storage solutions. In addition, all operations are performed locally so most processing tasks will execute relatively fast. Visualization in the sequence browser is also very fast and responsive since the system does not have to wait for individual data segments to load from a server. This means that the tool has not been designed primarily to handle extremely large datasets (e.g. full genomes), although it is possible to apply it for genome-wide binding site predictions if sufficient memory is available. However, MotifLab is ideal for in-depth analysis of small to moderate datasets ranging from a single sequence to a few hundred (or even a few thousand) sequences, such as promoter sequences from groups of co-expressed genes. It is also very well suited for interactive, visual exploration of datasets and for rapid hypothesis testing.

## Conclusions

Although vast amounts of genomic annotation data are now available to researchers who study transcriptional regulation, it is not necessarily trivial to make good use of this data for people who are not skilled in bioinformatics programming. The MotifLab workbench presented in this paper was designed to make it simple for users to obtain relevant data for sequences they want to study and to use this information in combination with existing motif discovery tools in many different ways. The utility and versatility of MotifLab was demonstrated through three practical analysis cases.

## Availability and requirements

**Project name:** MotifLab

**Project home page:**http://www.motiflab.org

**Operating system(s):** MotifLab itself is OS-independent, but some external tools used by MotifLab for motif discovery etc. might only be available for some operating systems.

**Programming language:** Java 1.6

**Other requirements:** None

**License:** None

**Any restrictions to use by non-academics:** None

## Abbreviations

Acc: Accuracy; ASP: Average site performance; AUC: Area under the curve; bp: Base pair; CC: Matthews correlation coefficient; F: F-measure; FP: False positive; PC: Performance coefficient; PPV: Positive predictive value; ROC: Receiver operating characteristic; Sn: Sensitivity (nucleotide level); sSn: Sensitivity (site level); Sp: Specificity; TF: Transcription factor; TP: True positive; TSS: Transcription start site.

## Competing interests

The authors declare that they have no competing interests.

## Author’s contributions

KK designed and implemented the MotifLab software and drafted the manuscript. FD supervised the project. Both authors participated in the design and analysis of the examples presented in the paper, and both authors revised and approved the final manuscript.

## Supplementary Material

Additional file 1 **Supplementary methods and additional results.** This supplementary file contains detailed descriptions of the procedure to generate and analyse the datasets used in examples 1 and 2, as well as results for individual datasets from those examples. Click here for file

## References

[B1] Wasserman WW Sandelin A Applied bioinformatics for the identification of regulatory elements Nat Rev Genet 2004 5 276 287 10.1038/nrg1315 15131651

[B2] Tompa M Li N Bailey TL Church GM De Moor B Eskin E Favorov AV Frith MC Fu Y Kent WJ Assessing computational tools for the discovery of transcription factor binding sites Nat Biotechnol 2005 23 137 144 10.1038/nbt1053 15637633

[B3] Okumura T Makiguchi H Makita Y Yamashita R Nakai K Melina II: a web tool for comparisons among several predictive algorithms to find potential motifs from promoter regions Nucleic Acids Res 2007 35 W227 W231 10.1093/nar/gkm362 17537821 PMC1933176

[B4] Sun H Yuan Y Wu Y Liu H Liu JS Xie H Tmod: toolbox of motif discovery Bioinformatics 2010 26 405 407 10.1093/bioinformatics/btp681 20007740 PMC2815662

[B5] Hu J Yang YD Kihara D EMD: an ensemble algorithm for discovering regulatory motifs in DNA sequences BMC Bioinforma 2006 7 342 10.1186/1471-2105-7-342 PMC1539026 16839417

[B6] Wijaya E Yiu SM Son NT Kanagasabai R Sung WK MotifVoter: a novel ensemble method for fine-grained integration of generic motif finders Bioinformatics 2008 24 2288 2295 10.1093/bioinformatics/btn420 18697768

[B7] Klepper K Sandve GK Abul O Johansen J Drablos F Assessment of composite motif discovery methods BMC Bioinforma 2008 9 123 10.1186/1471-2105-9-123 PMC2311304 18302777

[B8] ENCODE Project Consortium An integrated encyclopedia of DNA elements in the human genome Nature 2012 489 57 74 10.1038/nature11247 22955616 PMC3439153

[B9] Won KJ Ren B Wang W Genome-wide prediction of transcription factor binding sites using an integrated model Genome Biol 2010 11 R7 10.1186/gb-2010-11-1-r7 20096096 PMC2847719

[B10] Pique-Regi R Degner JF Pai AA Gaffney DJ Gilad Y Pritchard JK Accurate inference of transcription factor binding from DNA sequence and chromatin accessibility data Genome Res 2011 21 447 455 10.1101/gr.112623.110 21106904 PMC3044858

[B11] Lahdesmaki H Rust AG Shmulevich I Probabilistic inference of transcription factor binding from multiple data sources PLoS One 2008 3 e1820 10.1371/journal.pone.0001820 18364997 PMC2268002

[B12] Kuttippurathu L Hsing M Liu Y Schmidt B Maskell DL Lee K He A Pu WT Kong SW CompleteMOTIFs: DNA motif discovery platform for transcription factor binding experiments Bioinformatics 2011 27 715 717 10.1093/bioinformatics/btq707 21183585 PMC3105477

[B13] Kang K Kim J Chung JH Lee D Decoding the genome with an integrative analysis tool: combinatorial CRM Decoder Nucleic Acids Res 2011 39 e116 10.1093/nar/gkr516 21724599 PMC3177223

[B14] Herrmann C Van de Sande B Potier D Aerts S i-cisTarget: an integrative genomics method for the prediction of regulatory features and cis-regulatory modules Nucleic Acids Res 2012 40 e114 10.1093/nar/gks543 22718975 PMC3424583

[B15] Aerts S Van Loo P Thijs G Mayer H de Martin R Moreau Y De Moor B TOUCAN 2: the all-inclusive open source workbench for regulatory sequence analysis Nucleic Acids Res 2005 33 W393 W396 10.1093/nar/gki354 15980497 PMC1160115

[B16] Homann OR Johnson AD MochiView: versatile software for genome browsing and DNA motif analysis BMC Biol 2010 8 49 10.1186/1741-7007-8-49 20409324 PMC2867778

[B17] Hu Z Frith M Niu T Weng Z SeqVISTA: a graphical tool for sequence feature visualization and comparison BMC Bioinforma 2003 4 1 10.1186/1471-2105-4-1 PMC140037 12513700

[B18] Thomas-Chollier M Defrance M Medina-Rivera A Sand O Herrmann C Thieffry D van Helden J RSAT 2011: regulatory sequence analysis tools Nucleic Acids Res 2011 39 W86 W91 10.1093/nar/gkr377 21715389 PMC3125777

[B19] Dreszer TR Karolchik D Zweig AS Hinrichs AS Raney BJ Kuhn RM Meyer LR Wong M Sloan CA Rosenbloom KR The UCSC Genome Browser database: extensions and updates 2011 Nucleic Acids Res 2012 40 D918 D923 10.1093/nar/gkr1055 22086951 PMC3245018

[B20] Dowell RD Jokerst RM Day A Eddy SR Stein L The distributed annotation system BMC Bioinforma 2001 2 7 10.1186/1471-2105-2-7 PMC58584 11667947

[B21] Matys V Kel-Margoulis OV Fricke E Liebich I Land S Barre-Dirrie A Reuter I Chekmenev D Krull M Hornischer K TRANSFAC and its module TRANSCompel: transcriptional gene regulation in eukaryotes Nucleic Acids Res 2006 34 D108 D110 10.1093/nar/gkj143 16381825 PMC1347505

[B22] Portales-Casamar E Thongjuea S Kwon AT Arenillas D Zhao X Valen E Yusuf D Lenhard B Wasserman WW Sandelin A JASPAR 2010: the greatly expanded open-access database of transcription factor binding profiles Nucleic Acids Res 2010 38 D105 D110 10.1093/nar/gkp950 19906716 PMC2808906

[B23] Spivak AT Stormo GD ScerTF: a comprehensive database of benchmarked position weight matrices for Saccharomyces species Nucleic Acids Res 2012 40 D162 D168 10.1093/nar/gkr1180 22140105 PMC3245033

[B24] Hughes JD Estep PW Tavazoie S Church GM Computational identification of cis-regulatory elements associated with groups of functionally related genes in Saccharomyces cerevisiae J Mol Biol 2000 296 1205 1214 10.1006/jmbi.2000.3519 10698627

[B25] Liu X Brutlag DL Liu JS BioProspector: discovering conserved DNA motifs in upstream regulatory regions of co-expressed genes Pac Symp Biocomput 2001 6 127 138 11262934

[B26] Liu XS Brutlag DL Liu JS An algorithm for finding protein-DNA binding sites with applications to chromatin-immunoprecipitation microarray experiments Nat Biotechnol 2002 20 835 839 12101404 10.1038/nbt717

[B27] Bailey TL Elkan C Fitting a mixture model by expectation maximization to discover motifs in biopolymers Proc Int Conf Intell Syst Mol Biol 1994 2 28 36 7584402

[B28] Thijs G Lescot M Marchal K Rombauts S De Moor B Rouze P Moreau Y A higher-order background model improves the detection of promoter regulatory elements by Gibbs sampling Bioinformatics 2001 17 1113 1122 10.1093/bioinformatics/17.12.1113 11751219

[B29] Pavesi G Mauri G Pesole G An algorithm for finding signals of unknown length in DNA sequences Bioinformatics 2001 17 Suppl 1 S207 S214 10.1093/bioinformatics/17.suppl_1.S207 11473011

[B30] Gordan R Narlikar L Hartemink AJ Finding regulatory DNA motifs using alignment-free evolutionary conservation information Nucleic Acids Res 2010 38 e90 10.1093/nar/gkp1166 20047961 PMC2847231

[B31] Narlikar L Gordan R Hartemink AJ A nucleosome-guided map of transcription factor binding sites in yeast PLoS Comput Biol 2007 3 e215 10.1371/journal.pcbi.0030215 17997593 PMC2065891

[B32] Gordan R Hartemink AJ Using DNA duplex stability information for transcription factor binding site discovery Pac Symp Biocomput 2008 13 453 464 18229707

[B33] Cuellar-Partida G Buske FA McLeay RC Whitington T Noble WS Bailey TL Epigenetic priors for identifying active transcription factor binding sites Bioinformatics 2012 28 56 62 10.1093/bioinformatics/btr614 22072382 PMC3244768

[B34] Ernst J Plasterer HL Simon I Bar-Joseph Z Integrating multiple evidence sources to predict transcription factor binding in the human genome Genome Res 2010 20 526 536 10.1101/gr.096305.109 20219943 PMC2847756

[B35] Narlikar L Gordan R Ohler U Hartemink AJ Informative priors based on transcription factor structural class improve de novo motif discovery Bioinformatics 2006 22 e384 e392 10.1093/bioinformatics/btl251 16873497

[B36] Bailey TL Boden M Whitington T Machanick P The value of position-specific priors in motif discovery using MEME BMC Bioinforma 2010 11 179 10.1186/1471-2105-11-179 PMC2868008 20380693

[B37] Grant CE Bailey TL Noble WS FIMO: scanning for occurrences of a given motif Bioinformatics 2011 27 1017 1018 10.1093/bioinformatics/btr064 21330290 PMC3065696

[B38] Kulakovskiy IV Boeva VA Favorov AV Makeev VJ Deep and wide digging for binding motifs in ChIP-Seq data Bioinformatics 2010 26 2622 2623 10.1093/bioinformatics/btq488 20736340

[B39] Carvalho AM Oliveira AL GRISOTTO: A greedy approach to improve combinatorial algorithms for motif discovery with prior knowledge Algorithms Mol Biol 2011 6 13 10.1186/1748-7188-6-13 21513505 PMC3112114

[B40] Klepper K Drablos F PriorsEditor: a tool for the creation and use of positional priors in motif discovery Bioinformatics 2010 26 2195 2197 10.1093/bioinformatics/btq357 20628076 PMC2922893

[B41] Sandve GK Abul O Walseng V Drablos F Improved benchmarks for computational motif discovery BMC Bioinforma 2007 8 193 10.1186/1471-2105-8-193 PMC1903367 17559676

[B42] Aerts S Van Loo P Thijs G Moreau Y De Moor B Computational detection of cis-regulatory modules Bioinformatics 2003 19 Suppl 2 ii5 ii14 10.1093/bioinformatics/btg1052 14534164

[B43] Seamon KB Padgett W Daly JW Forskolin: unique diterpene activator of adenylate cyclase in membranes and in intact cells Proc Natl Acad Sci USA 1981 78 3363 3367 10.1073/pnas.78.6.3363 6267587 PMC319568

[B44] Mantovani R The molecular biology of the CCAAT-binding factor NF-Y Gene 1999 239 15 27 10.1016/S0378-1119(99)00368-6 10571030

[B45] Zhong ZD Hammani K Bae WS DeClerck YA NF-Y and Sp1 cooperate for the transcriptional activation and cAMP response of human tissue inhibitor of metalloproteinases-2 J Biol Chem 2000 275 18602 18610 10.1074/jbc.M001389200 10764764

[B46] Cote F Schussler N Boularand S Peirotes A Thevenot E Mallet J Vodjdani G Involvement of NF-Y and Sp1 in basal and cAMP-stimulated transcriptional activation of the tryptophan hydroxylase (TPH) gene in the pineal gland J Neurochem 2002 81 673 685 10.1046/j.1471-4159.2002.00890.x 12065627

[B47] De Rasmo D Signorile A Papa F Roca E Papa S cAMP/Ca2+ response element-binding protein plays a central role in the biogenesis of respiratory chain proteins in mammalian cells IUBMB Life 2010 62 447 452 20503437 10.1002/iub.342

