# Supplementary material for: MotifLab: a tools and data integration workbench for motif discovery and regulatory sequence analysis
Source: BMC Bioinformatics. 2013 Jan 16;14:9. doi: 10.1186/1471-2105-14-9 (PMC3556059; doi:10.1186/1471-2105-14-9)
Supplement: Additional file 1 — Supplementary methods and additional results. This supplementary file contains detailed descriptions of the procedure to generate and analyse the datasets used in examples 1 and 2, as well as results for individual datasets from those examples. [file 1471-2105-14-9-S1.pdf]

Supplementary material

MotifLab: a tools and data integration workbench for motif discovery and regulatory sequence analysis

Kjetil Klepper and Finn Drabløs

Department of Cancer Research and Molecular Medicine, Norwegian University of Science and Technology, Trondheim, Norway

Email addresses:

KK: kjetil.klepper@ntnu.no

FD: finn.drablos@ntnu.no

Example 1: Using automatically generated positional priors to improve motif discovery with MEME

The 22 datasets we used for the first example case were based on the "algorithm_real" version of the single motif discovery benchmark suite described in Sandve *et al.* [1]. This benchmark consisted of 50 datasets based on transcription factor binding sites annotated in the TRANSFAC database. Each of the datasets was based on a single TRANSFAC motif model and the binding sites that went into constructing this model. In the "algorithm_real" datasets the binding sites were set in their original genomic sequence context.

In order to be able to include several different features in our positional priors, we had to limit the data to sequences originating from either human or mouse. To find the genomic coordinates for each sequence, we used BLAT [2] to map the sequences to human genome build "hg18" and mouse build "mm9" respectively. Sequences that could not be mapped to these genome builds in a unique and satisfactory way were discarded. Because there could be variation (SNPs and indels) between the original benchmark sequences and the hg18 and mm9 builds, we manually verified that all the target binding sites could still be found in the sequences by scanning with the PWM for the dataset. Sequences where the target binding site could not be found were discarded.

Many of the benchmark datasets were actually based on the same (or very similar) transcription factors but represented by different TRANSFAC motif models. Because of this there was some overlap in the binding sites between the datasets in the original benchmark. Since we wanted to use leave-one-out cross-validation to validate our approach for automatic generation of positional priors, we wanted to make sure that there was no overlap between the sequences in the datasets we used. Datasets for similar TFs that had overlapping binding sites were therefore combined together. We also compared the genomic coordinates of all the sequences to see if there was still any overlap due to binding sites for different transcription factors being located close together in the same promoters. Sequences that still overlapped with others were cropped to remove the overlap, and where possible we also tried to extend the cropped sequences in the opposite direction to restore them to their original lengths. Next, we tried to rebuild the PWM for each dataset based on the remaining binding sites, and in a few cases where the PWM had obvious flanks with very low information content the PWM and corresponding binding site annotations were cropped to cover just the core regions. Finally, we required that each of the datasets should contain at least 5 sequences, and this left us with only 22 remaining datasets.

The feature datasets used for the positional priors were all based on data available from the USCS Genome Browser [3]. The conservation track was based on "phastCons44way" (hg18) and "phastCons30way" (mm9). The "conservation peaks" feature was derived from the conservation track using MotifLab's "apply" operation with the "peaks" window function. This function used a 30bp sliding window centered on each position

and divided the window into three segments spanning respectively the leftmost 40% of the window, the middle 20% and the rightmost 40%. The function then found the smallest track-value in the leftmost and rightmost segments, and unless the highest of these values was less than 10% of the value in the center position of the window, the center value would be set to 0. This meant that only narrow peaks in the conservation track (where the value was much higher than values on either side) would be retained, and long stretches with similar values would be set to 0. The DNaseHS peaks, H3K4me1 and H3K4me3 tracks were based on peak regions from many different cell types, and the TFBS ChIP-seq track was based on data for many different transcription factors.

The Priors Generators we used were based on a neural network with 10 input nodes corresponding to the 10 input features, 4 nodes in a hidden layer and a single output node. In the cross-validation approach we held out one of the 22 datasets and build a training set for each Priors Generator based on the other 21 datasets using all positions within binding sites as positive training examples and 4000 negative examples sampled at equal distance intervals from the background sequence. Duplicate training examples with identical values for all features were discarded before the network was trained for 1000 epochs. Values for numeric features were fed directly to the input nodes, but for region datasets we used a value of 1 for positions inside the regions and 0 for positions outside. After the network was trained we used MotifLab's "predict" operation to generate positional priors values for each sequence position in the dataset that was held out. The value for each position was directly based on the value of the output node in the network. This value reflected the Priors Generator's belief that the position could be located *inside* a binding site, but MEME interprets the value of each position in a priors track as the probability that a binding site could *start* at that position. We therefore used MotifLab's "apply" operation to apply a sliding "sum" window function which set the value at each position in the raw priors track to the sum of the values for the next 10bp starting at that position. Since MEME does not allow a priors track to have the value 0 for any positions, we also used the "normalize" operation to normalize the value range in each sequence from [0, *max*] to [0.02, *max*] (where *max* was the maximum value in each sequence). Finally, we normalized the values within each sequence so that they summed to 1.0. For the positional priors based solely on conservation, we used the original conservation track as the "raw" priors and applied the 10bp "sum" window and normalized the track in the same way as we did for the automatically generated priors.

MEME was run in OOPS-mode to search for exactly one occurrence of the target motif per sequence with a minimum motif length of 8bp and a maximum length of 16bp.

The ROC-curves in Figure 4 were based on the raw values of the feature tracks (without applying the 10bp “sum” window and normalizations). The combined priors used for that figure were generated by a “typical” trained Priors Generator for each dataset. The “typical” Priors Generator would be the one whose results were closest to the average, median or mode over the 20 runs. Supplementary Tables 1 and 2 provide justifications for choosing the “typical” Priors Generator for each dataset.

Supplementary Table 1)

The table shows the CC-scores for each individual dataset in all 20 runs when MEME was guided by the auto-generated positional priors.

Dataset	1	2	3	4	5	6	7	8	9	10	11	12	13	14	15	16	17	18	19	20
M00621	-0,012	-0,010	-0,011	-0,009	-0,009	-0,009	-0,011	0,094	0,002	-0,009	0,024	0,113	-0,012	0,376	0,024	0,147	0,147	0,016	-0,012	0,007
M00622	-0,038	0,086	-0,042	E	-0,042	-0,038	-0,042	-0,042	-0,042	-0,046	-0,046	0,086	-0,036	0,127	-0,042	-0,042	-0,036	-0,042	-0,038	-0,042
M00658	0,328	0,664	0,010	0,664	0,269	0,302	0,205	0,420	0,312	-0,009	0,312	0,139	0,664	0,312	0,312	0,312	-0,008	0,664	0,420	-0,008
M00699	0,471	0,174	0,174	0,174	0,174	0,174	0,174	0,174	0,174	0,174	0,174	0,174	0,381	0,174	0,174	0,174	0,446	0,174	0,174	0,102
M00733	0,245	0,296	0,120	0,245	0,259	0,114	0,259	0,259	0,259	0,245	0,272	0,166	0,259	0,259	0,259	0,259	0,284	0,245	0,259	0,259
M00764	-0,011	-0,013	-0,011	-0,009	-0,012	-0,011	-0,011	-0,011	-0,013	-0,013	-0,011	-0,009	-0,012	-0,010	-0,012	-0,013	-0,011	-0,011	-0,013	-0,011
M00771	0,344	0,344	0,344	0,344	0,505	0,344	0,389	0,344	0,344	0,344	0,229	-0,009	0,345	0,344	0,344	0,479	0,462	0,462	0,344	0,344
M00774	0,506	-0,009	0,899	0,593	0,846	0,846	-0,009	-0,009	0,846	0,024	0,539	0,539	-0,011	-0,009	0,846	0,846	0,447	0,593	0,066	0,447
M00797	0,310	0,315	0,080	0,155	0,070	0,415	0,415	0,149	0,175	0,315	0,315	0,129	0,162	0,315	0,513	0,415	0,217	0,313	0,415	0,310
M00799	-0,010	0,038	-0,010	-0,010	-0,008	0,038	-0,010	-0,010	0,038	-0,010	-0,008	0,394	-0,008	-0,010	-0,010	0,294	-0,009	-0,008	-0,009	-0,010
M00809	-0,006	-0,008	-0,007	-0,007	-0,007	-0,008	-0,006	-0,008	-0,006	-0,008	-0,006	-0,006	-0,006	-0,007	-0,007	-0,007	-0,006	-0,007	-0,007	-0,006
M00920	-0,010	-0,010	0,693	-0,010	-0,010	-0,012	0,693	-0,010	-0,010	-0,010	-0,010	-0,010	-0,010	-0,012	-0,012	-0,010	0,693	-0,010	-0,012	-0,010
M00929	0,233	0,295	-0,010	0,233	0,233	0,233	0,360	-0,011	0,360	0,233	0,233	0,360	-0,004	0,377	0,235	0,233	0,504	0,228	0,360	0,377
M00965	-0,011	0,065	0,130	0,130	-0,011	-0,008	-0,011	0,130	-0,011	0,065	0,130	0,130	0,061	-0,011	0,065	0,130	0,130	-0,009	0,130	-0,011
M00978	0,105	0,105	-0,015	-0,014	0,030	0,105	-0,014	0,105	0,105	0,105	0,228	0,105	0,109	0,219	0,105	0,219	0,105	-0,014	-0,014	0,105
M00982	0,616	0,548	0,516	0,358	0,548	0,548	0,459	0,459	0,622	0,217	0,536	0,260	0,288	0,284	0,548	0,622	0,459	-0,004	0,622	0,579
M00983	0,685	0,568	0,568	0,590	0,685	0,590	0,546	0,568	0,568	0,685	0,568	0,685	0,677	0,568	0,723	0,677	0,685	0,685	0,685	0,685
M01007	0,879	0,624	0,879	0,879	0,879	0,879	0,879	0,879	-0,012	0,879	0,007	0,006	0,879	0,879	0,879	0,879	0,879	0,879	0,879	0,879
M01011	0,487	0,453	0,415	0,487	0,372	0,415	0,401	0,366	0,503	0,401	0,470	0,305	0,386	0,453	0,429	0,401	0,487	0,415	0,366	0,401
M01035	0,083	0,083	0,088	-0,008	0,083	-0,009	0,083	0,088	-0,008	-0,009	-0,010	-0,008	-0,007	0,083	0,083	-0,008	0,083	0,083	0,088	-0,008
M01036	0,167	-0,009	0,260	-0,012	-0,009	-0,012	0,116	0,178	0,167	0,260	-0,012	-0,012	0,148	-0,009	-0,009	0,345	-0,010	0,260	-0,009	-0,012
M01067	0,135	0,135	0,135	0,123	0,135	0,135	0,076	-0,007	0,146	0,135	0,135	0,076	0,135	0,135	0,146	0,076	0,048	0,125	0,135	-0,007

Supplementary Table 2)

The two first columns are CC-scores for each of the 22 datasets when MEME was guided by the uniform priors track and the conservation-based priors. The following three columns are the average, median and mode values from the 20 runs with the combined positional priors show in supplementary Table 1. The sixth column shows the CC-value which was selected as the “most typical” among the 20 runs based on its closeness to either the average, median or mode value. The last column is the number of the first run which has this typical CC-score for that dataset. CC-scores below 0.2 are colored red, scores between 0.2 and 0.4 are colored yellow and scores above 0.4 are colored green.

Uniform	Conservation	Average Combined	Median Combined	Mode Combined	Typical Combined	Dataset	Typical run
-0,011	-0,011	0,042	-0,004	-0,009	-0,009	M00621	4
-0,036	-0,036	-0,020	-0,042	-0,042	-0,042	M00622	3
-0,010	0,518	0,314	0,312	0,312	0,312	M00658	9
0,264	0,174	0,209	0,174	0,174	0,174	M00699	2
-0,023	-0,021	0,241	0,259	0,259	0,259	M00733	5
-0,012	0,158	-0,011	-0,011	-0,011	-0,011	M00764	1
-0,011	0,344	0,349	0,344	0,344	0,344	M00771	1
-0,013	0,367	0,442	0,522	0,846	0,506	M00774	1
-0,017	0,283	0,275	0,311	0,315	0,310	M00797	1
-0,010	-0,010	0,033	-0,009	-0,010	-0,010	M00799	1
-0,007	-0,006	-0,007	-0,007	-0,006	-0,007	M00809	3
-0,012	0,045	0,095	-0,010	-0,010	-0,010	M00920	1
-0,013	0,089	0,253	0,233	0,233	0,233	M00929	1
-0,011	-0,011	0,061	0,065	0,130	0,065	M00965	2
-0,020	-0,014	0,089	0,105	0,105	0,105	M00978	1
0,548	0,358	0,454	0,526	0,548	0,516	M00982	3
0,590	0,568	0,634	0,677	0,685	0,677	M00983	13
-0,012	-0,012	0,734	0,879	0,879	0,879	M01007	1
-0,011	0,453	0,421	0,415	0,401	0,415	M01011	3
-0,008	-0,007	0,043	0,083	0,083	0,083	M01035	1
-0,010	-0,012	0,089	-0,009	-0,009	-0,009	M01036	2
-0,010	-0,010	0,108	0,135	0,135	0,135	M01067	1

Figure S1a) Example 1: Combined results from all 22 datasets

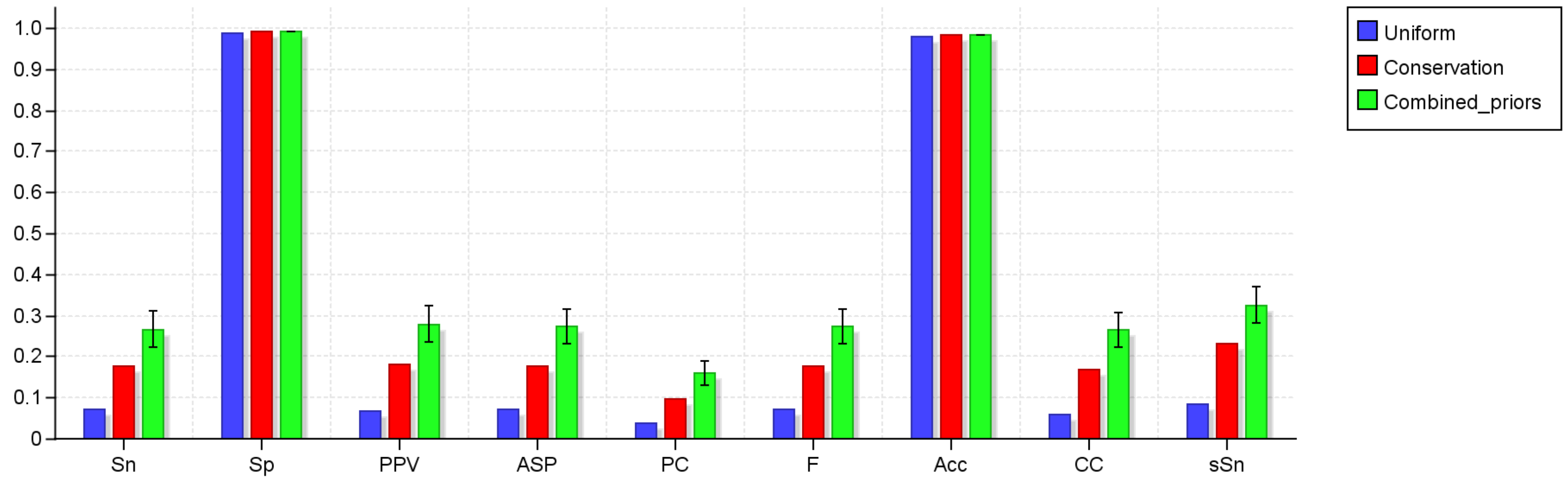

Track	Sn	Sp	PPV	ASP	PC	F	Acc	CC	sSn
**Uniform**	0.073	0.988	0.069	0.071	0.037	0.071	0.978	0.06	0.084
**Conservation**	0.175	0.991	0.182	0.178	0.098	0.178	0.981	0.169	0.229
**Combined_priors**	0.267	0.992	0.278	0.272	0.158	0.272	0.983	0.264	0.325

Figure S1b) Example 1: Dataset “M00621 – C/EBP delta”

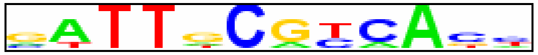

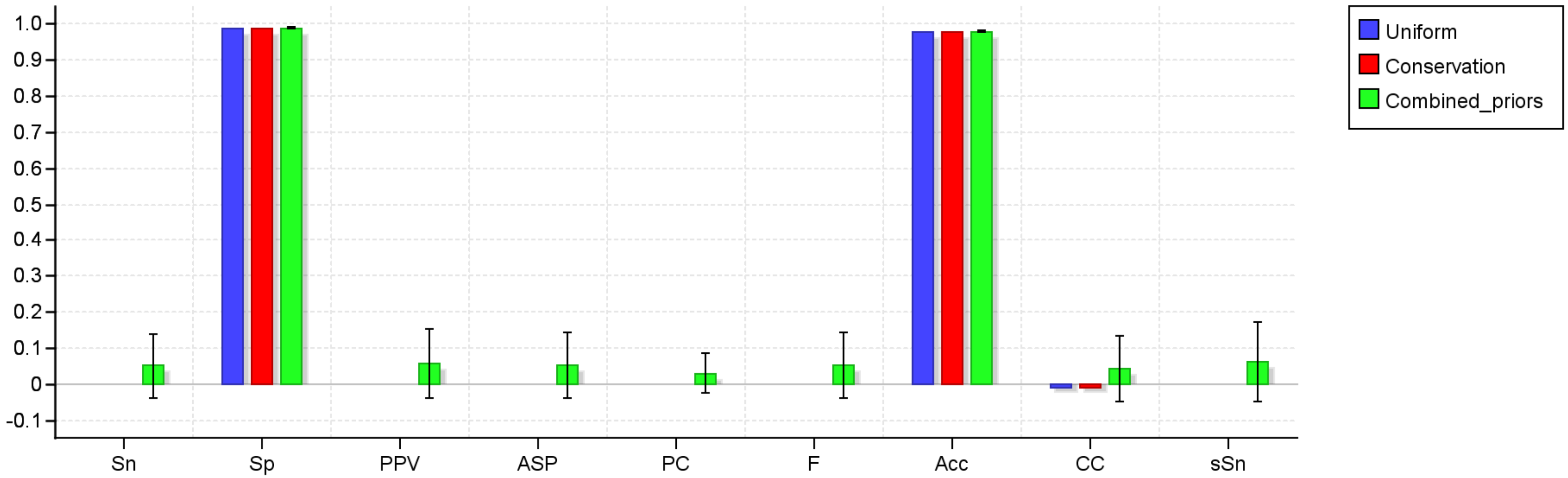

Track	Sn	Sp	PPV	ASP	PC	F	Acc	CC	sSn
**Uniform**	0	0.989	0	0	0	0	0.978	-0.011	0
**Conservation**	0	0.989	0	0	0	0	0.978	-0.011	0
**Combined_priors**	0.051	0.99	0.055	0.053	0.03	0.052	0.979	0.042	0.06

Figure S1c) Example 1: Dataset “M00622 – C/EBP gamma”

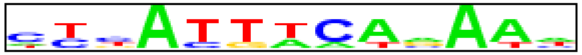

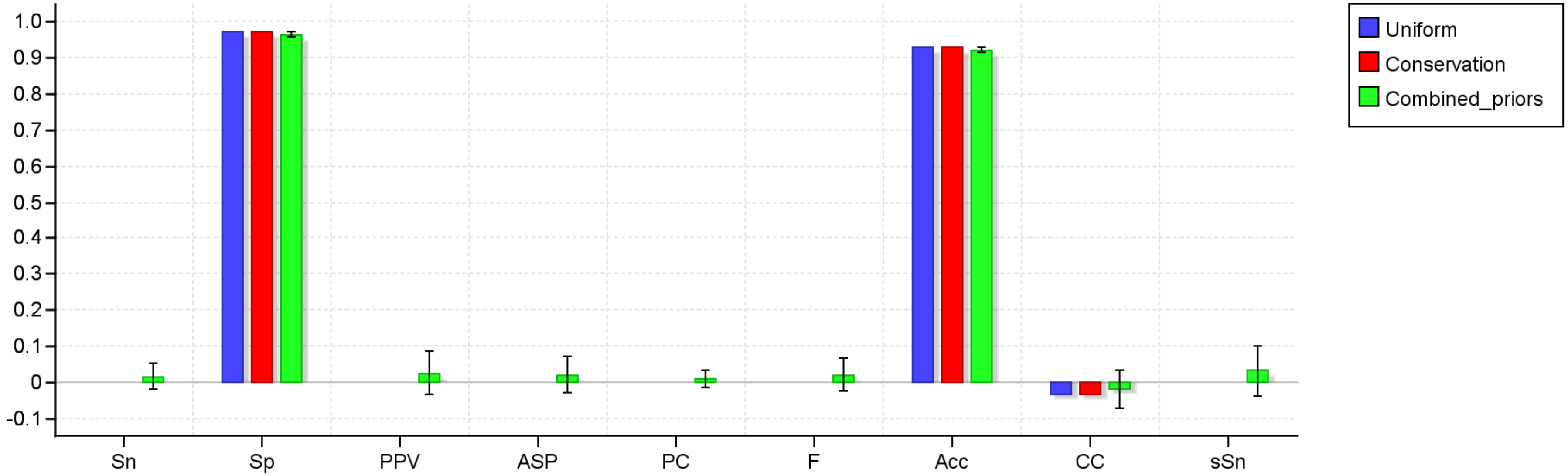

Track	Sn	Sp	PPV	ASP	PC	F	Acc	CC	sSn
**Uniform**	0	0.972	0	0	0	0	0.929	-0.036	0
**Conservation**	0	0.972	0	0	0	0	0.929	-0.036	0
**Combined_priors**	0.015	0.964	0.025	0.02	0.01	0.019	0.922	-0.02	0.03

Figure S1d) Example 1: Dataset "M00658 – PU.1"

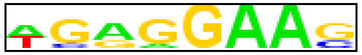

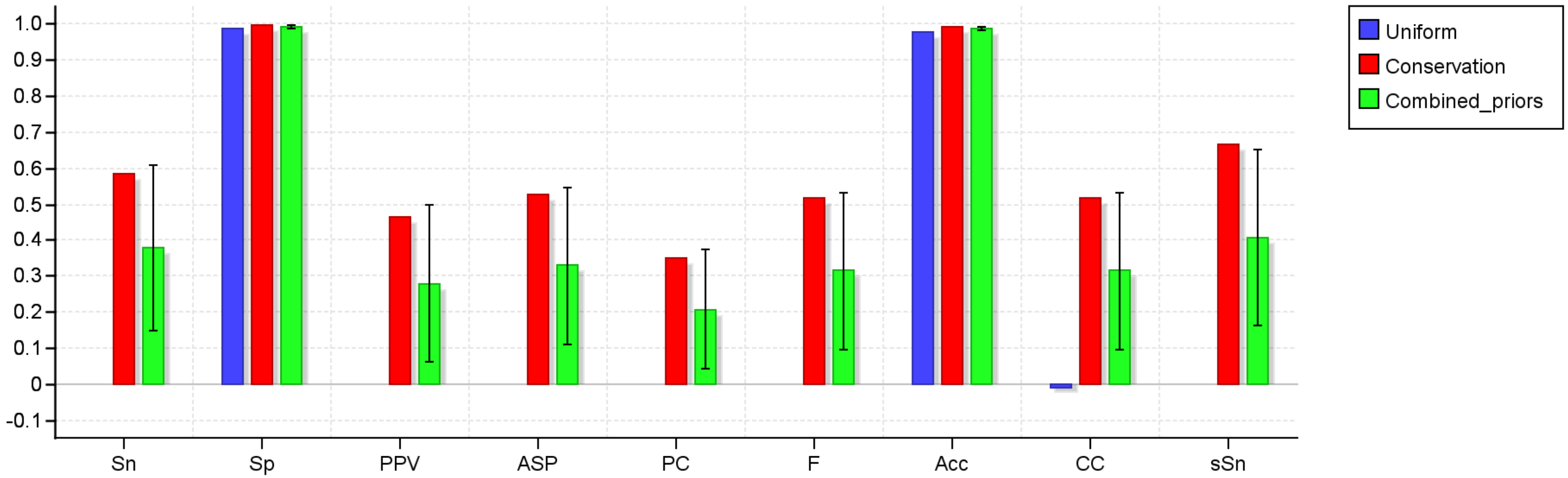

Track	Sn	Sp	PPV	ASP	PC	F	Acc	CC	sSn
**Uniform**	0	0.988	0	0	0	0	0.98	-0.01	0
**Conservation**	0.583	0.995	0.467	0.525	0.35	0.519	0.992	0.518	0.667
**Combined_priors**	0.377	0.992	0.279	0.328	0.207	0.313	0.987	0.314	0.408

Figure S1e) Example 1: Dataset “M00699 – ICSBP (IRF8)”

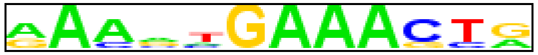

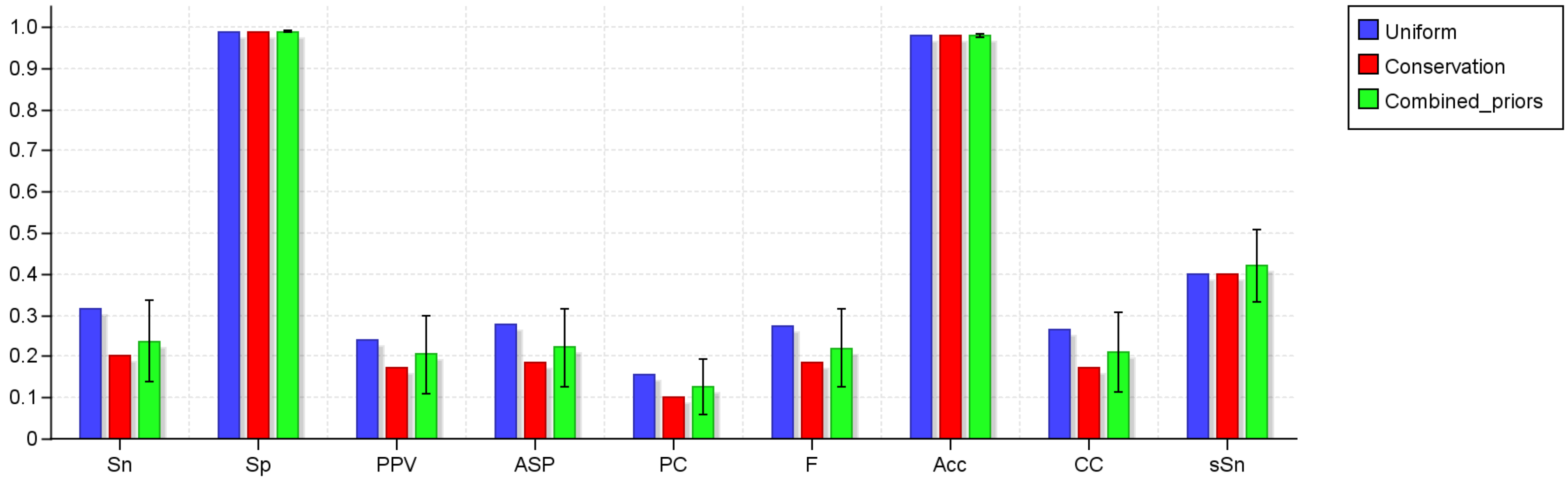

Track	Sn	Sp	PPV	ASP	PC	F	Acc	CC	sSn
**Uniform**	0.317	0.987	0.238	0.277	0.157	0.271	0.979	0.264	0.4
**Conservation**	0.2	0.988	0.171	0.186	0.102	0.185	0.978	0.174	0.4
**Combined_priors**	0.237	0.988	0.205	0.221	0.127	0.219	0.979	0.209	0.42

Figure S1f) Example 1: Dataset “M00733 – SMAD4”

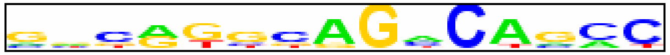

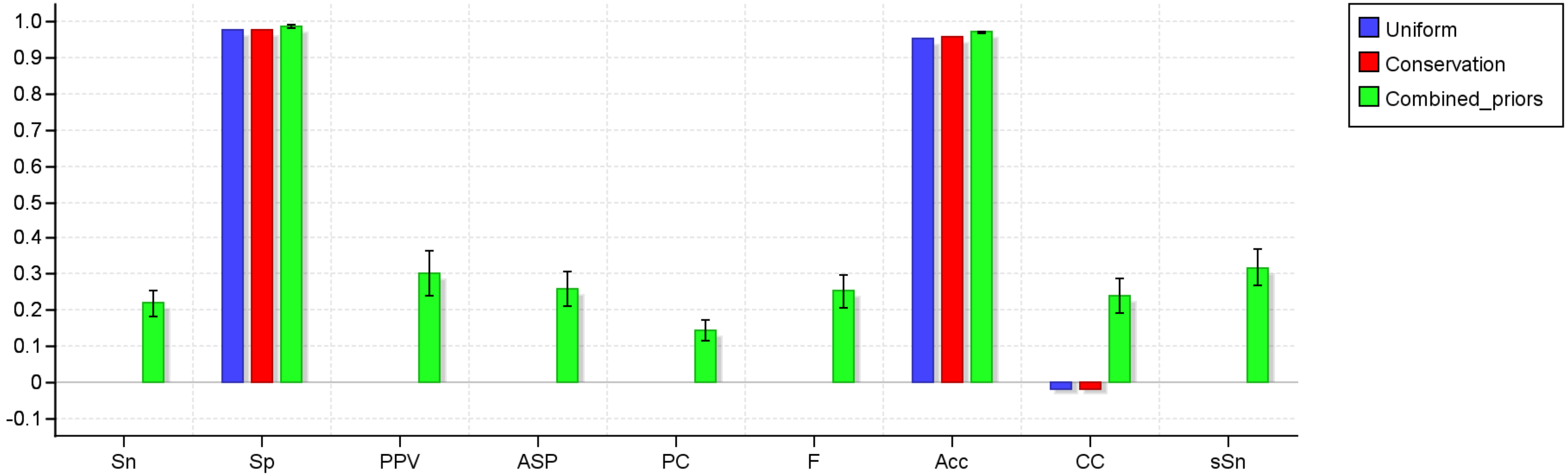

Track	Sn	Sp	PPV	ASP	PC	F	Acc	CC	sSn
**Uniform**	0	0.977	0	0	0	0	0.956	-0.023	0
**Conservation**	0	0.98	0	0	0	0	0.958	-0.021	0
**Combined_priors**	0.217	0.988	0.301	0.259	0.145	0.251	0.971	0.241	0.317

Figure S1g) Example 1: Dataset "M00764 – HNF-4 direct repeat 1"

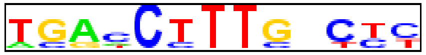

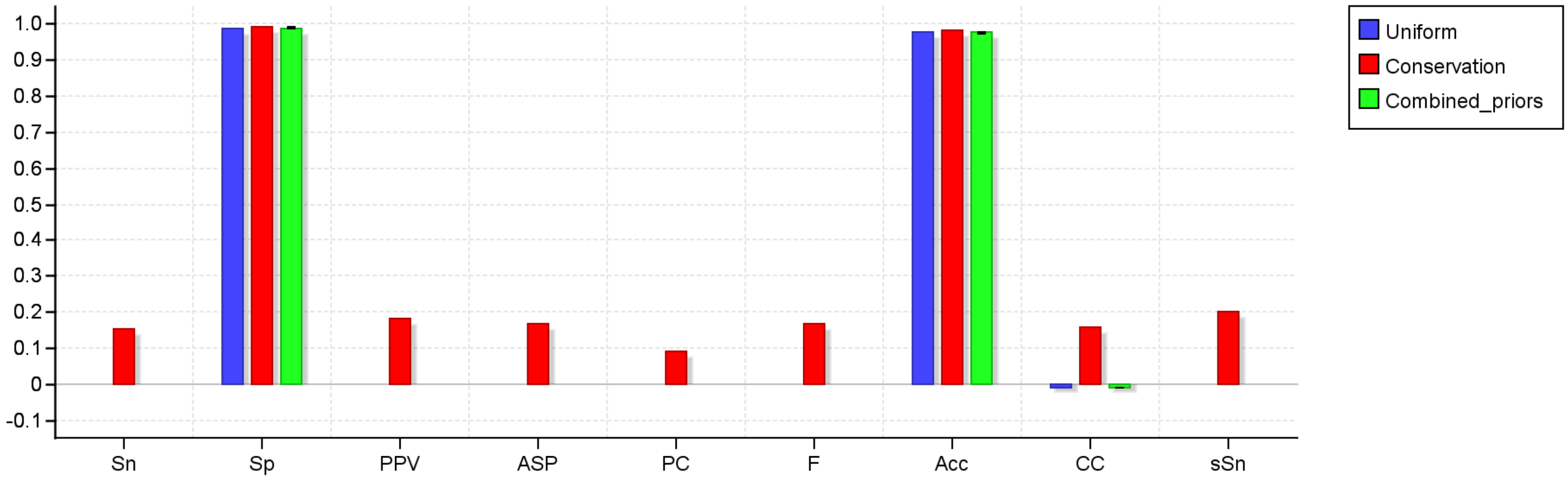

Track	Sn	Sp	PPV	ASP	PC	F	Acc	CC	sSn
**Uniform**	0	0.987	0	0	0	0	0.976	-0.012	0
**Conservation**	0.154	0.992	0.182	0.168	0.091	0.167	0.982	0.158	0.2
**Combined_priors**	0	0.989	0	0	0	0	0.978	-0.011	0

Figure S1h) Example 1: Dataset “M00771 –Ets”

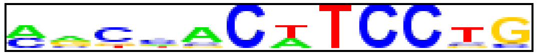

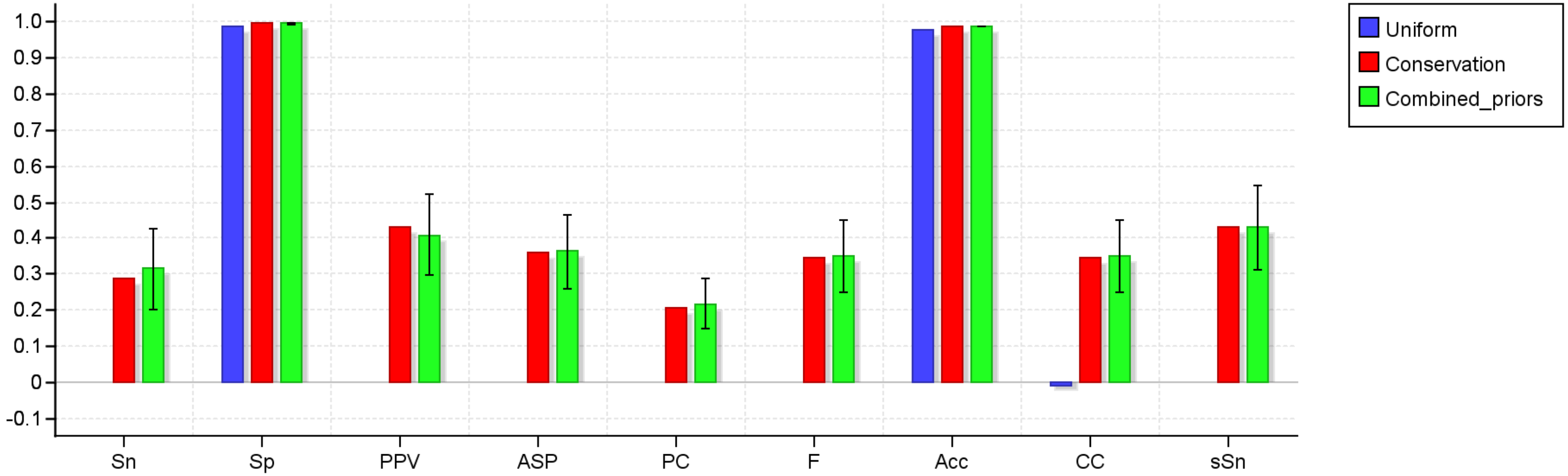

Track	Sn	Sp	PPV	ASP	PC	F	Acc	CC	sSn
**Uniform**	0	0.99	0	0	0	0	0.978	-0.011	0
**Conservation**	0.286	0.996	0.429	0.357	0.207	0.343	0.988	0.344	0.429
**Combined_priors**	0.314	0.995	0.409	0.361	0.216	0.35	0.987	0.349	0.429

Figure S1i) Example 1: Dataset “M00774 – NF-kB”

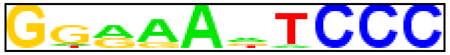

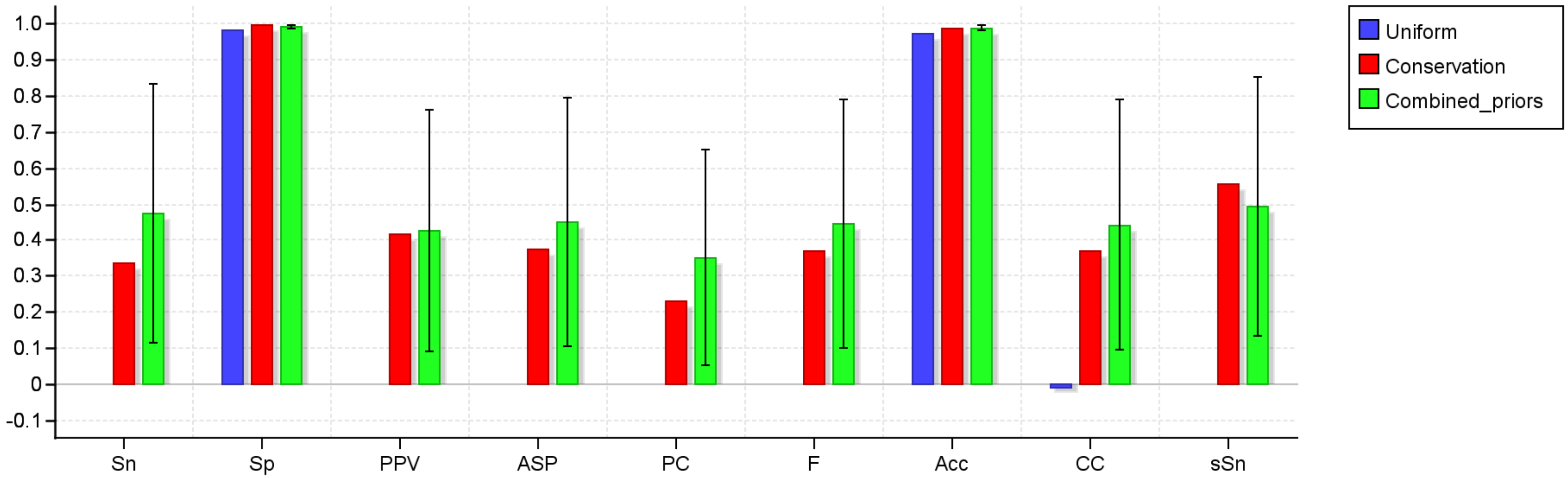

Track	Sn	Sp	PPV	ASP	PC	F	Acc	CC	sSn
**Uniform**	0	0.984	0	0	0	0	0.974	-0.013	0
**Conservation**	0.333	0.995	0.417	0.375	0.227	0.37	0.988	0.367	0.556
**Combined_priors**	0.474	0.994	0.426	0.45	0.351	0.445	0.988	0.442	0.494

Figure S1j) Example 1: Dataset “M00797 – HIF1 (hypoxia induced factor)”

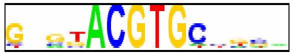

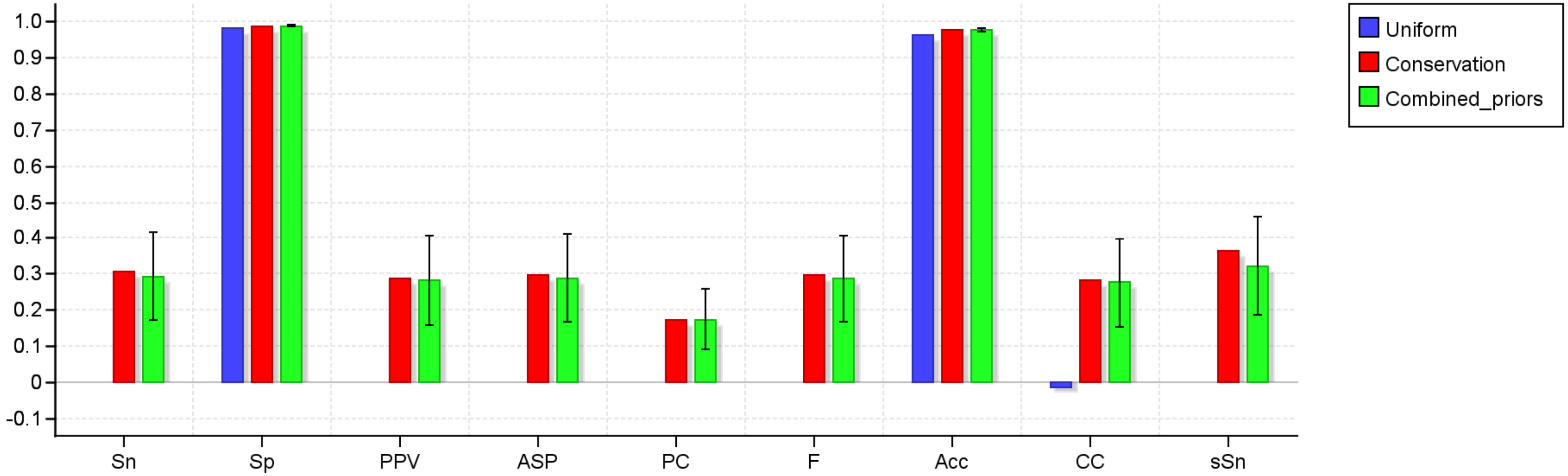

Track	Sn	Sp	PPV	ASP	PC	F	Acc	CC	sSn
**Uniform**	0	0.981	0	0	0	0	0.966	-0.017	0
**Conservation**	0.305	0.988	0.285	0.295	0.173	0.295	0.977	0.283	0.364
**Combined_priors**	0.294	0.988	0.281	0.288	0.173	0.286	0.977	0.275	0.323

Figure S1k) Example 1: Dataset "M00799 – MYC"

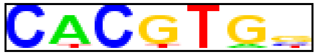

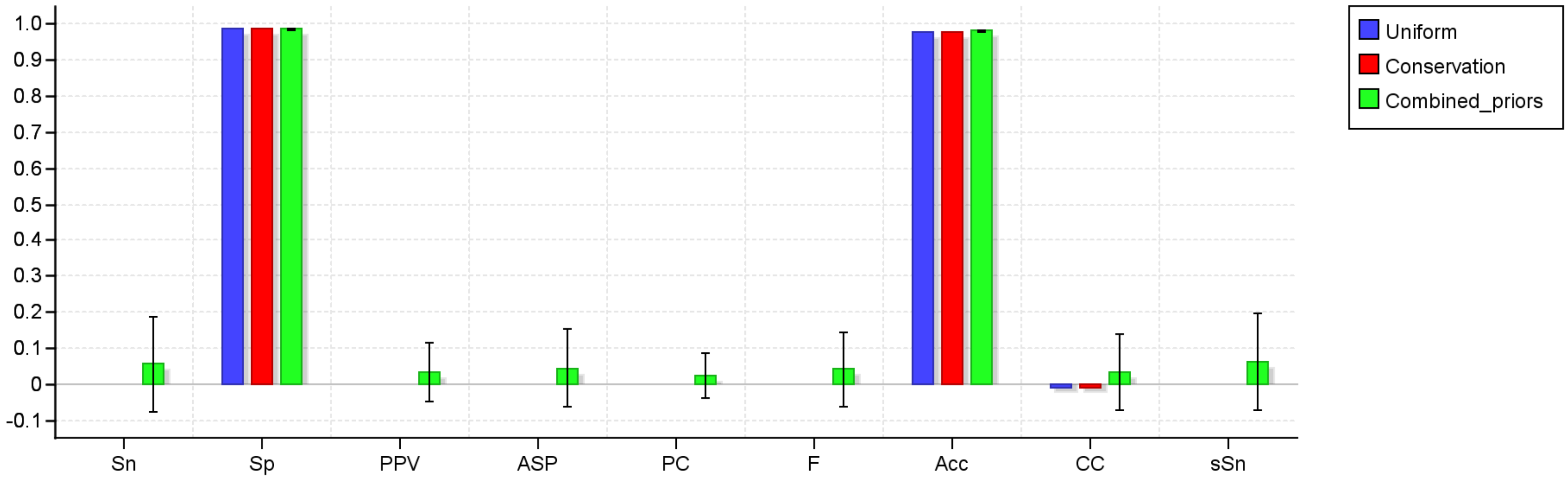

Track	Sn	Sp	PPV	ASP	PC	F	Acc	CC	sSn
**Uniform**	0	0.985	0	0	0	0	0.979	-0.01	0
**Conservation**	0	0.985	0	0	0	0	0.979	-0.01	0
**Combined_priors**	0.054	0.987	0.033	0.043	0.024	0.041	0.981	0.033	0.062

Figure S1l) Example 1: Dataset “M00809 – FOX factors”

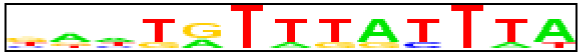

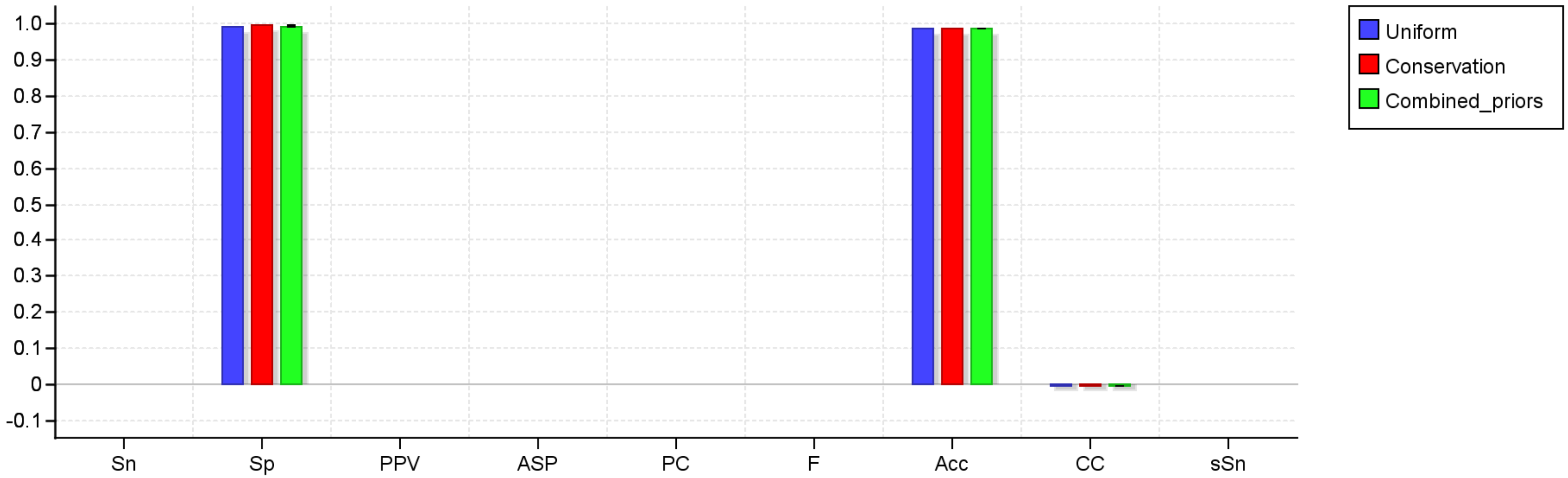

Track	Sn	Sp	PPV	ASP	PC	F	Acc	CC	sSn
**Uniform**	0	0.994	0	0	0	0	0.986	-0.007	0
**Conservation**	0	0.995	0	0	0	0	0.988	-0.006	0
**Combined_priors**	0	0.994	0	0	0	0	0.987	-0.007	0

Figure S1m) Example 1: Dataset “M00920 – E2F”

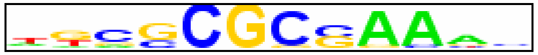

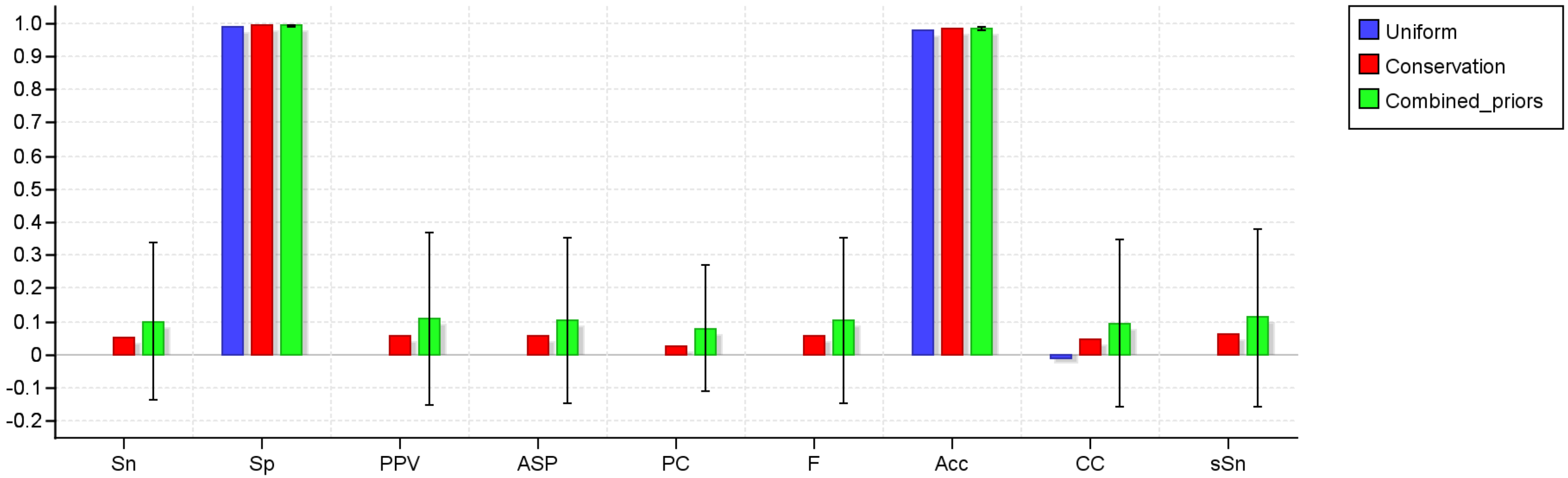

Track	Sn	Sp	PPV	ASP	PC	F	Acc	CC	sSn
**Uniform**	0	0.987	0	0	0	0	0.977	-0.012	0
**Conservation**	0.052	0.991	0.057	0.054	0.028	0.054	0.982	0.045	0.062
**Combined_priors**	0.1	0.991	0.109	0.105	0.08	0.104	0.982	0.095	0.112

Figure S1n) Example 1: Dataset “M00929 – MyoD”

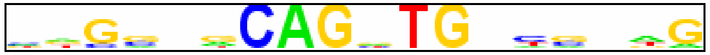

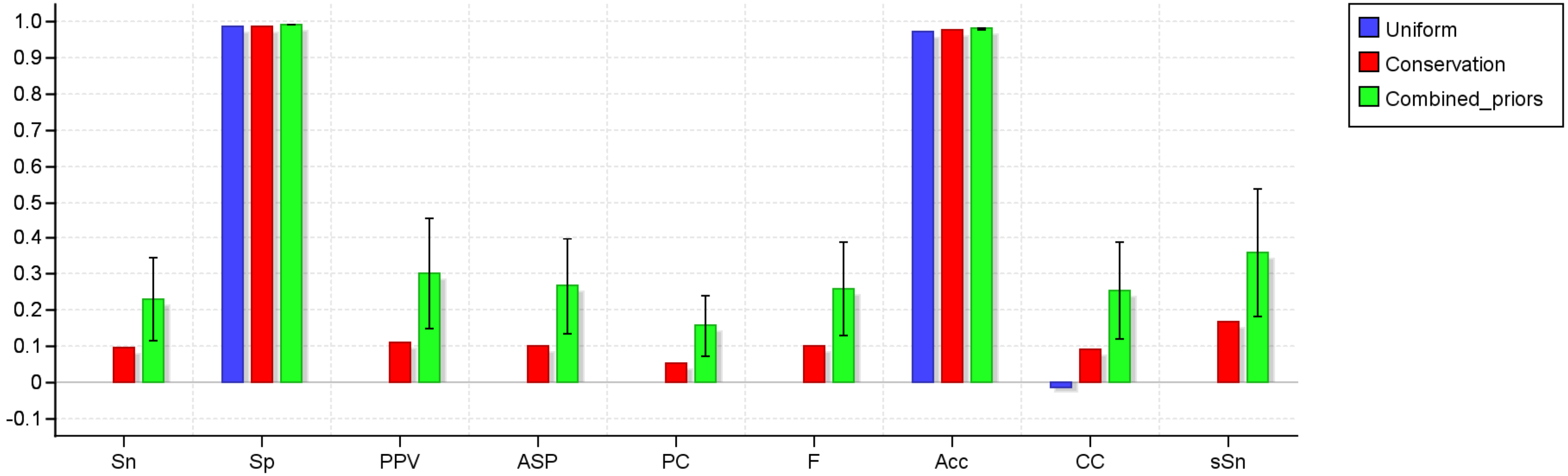

Track	Sn	Sp	PPV	ASP	PC	F	Acc	CC	sSn
**Uniform**	0	0.988	0	0	0	0	0.973	-0.013	0
**Conservation**	0.093	0.989	0.111	0.102	0.053	0.101	0.976	0.089	0.167
**Combined_priors**	0.23	0.992	0.301	0.266	0.155	0.259	0.981	0.253	0.358

Figure S1o) Example 1: Dataset “M00965 – DR4 (direct repeat 4)”

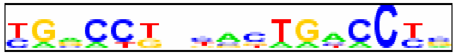

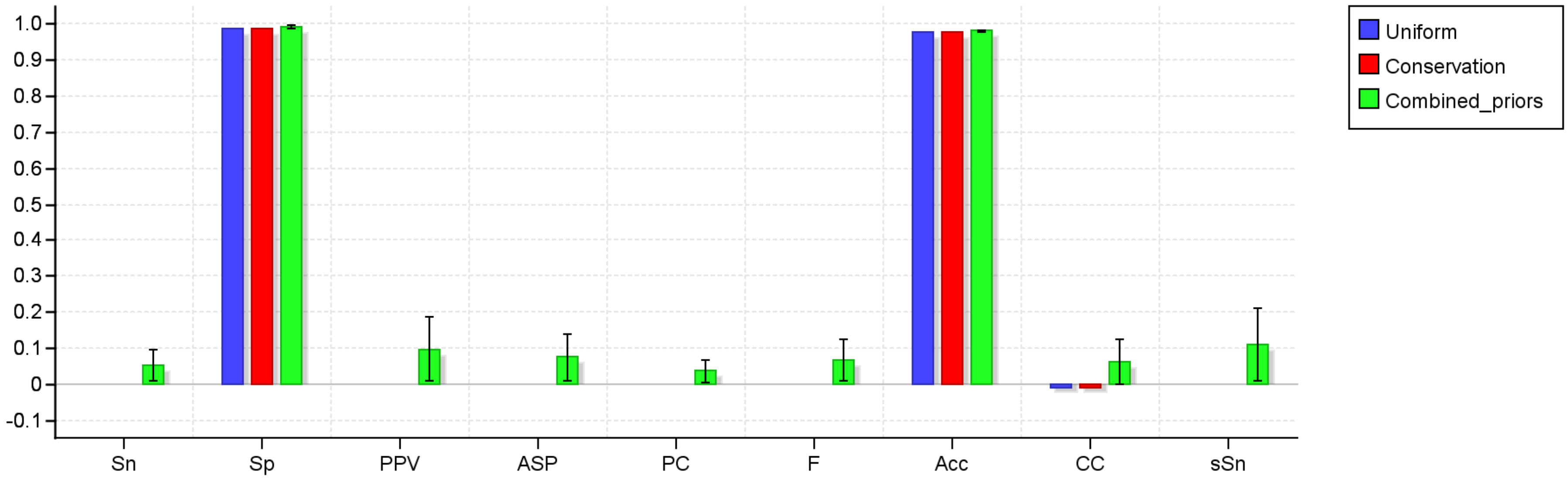

Track	Sn	Sp	PPV	ASP	PC	F	Acc	CC	sSn
**Uniform**	0	0.99	0	0	0	0	0.978	-0.011	0
**Conservation**	0	0.99	0	0	0	0	0.978	-0.011	0
**Combined_priors**	0.051	0.993	0.097	0.074	0.035	0.066	0.982	0.061	0.11

Figure S1p) Example 1: Dataset “M00978 – EF1, TCF1”

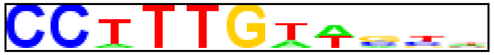

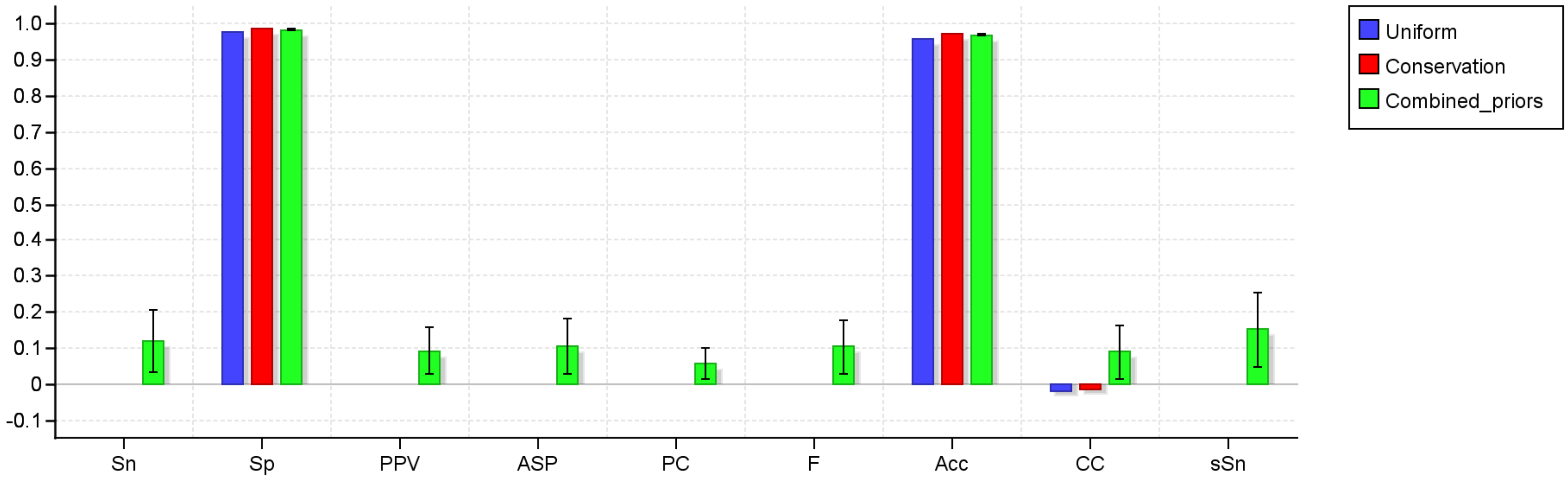

Track	Sn	Sp	PPV	ASP	PC	F	Acc	CC	sSn
**Uniform**	0	0.976	0	0	0	0	0.961	-0.02	0
**Conservation**	0	0.988	0	0	0	0	0.973	-0.014	0
**Combined_priors**	0.119	0.983	0.092	0.105	0.056	0.104	0.97	0.089	0.15

Figure S1q) Example 1: Dataset "M00982 – KROX"

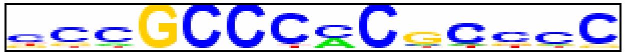

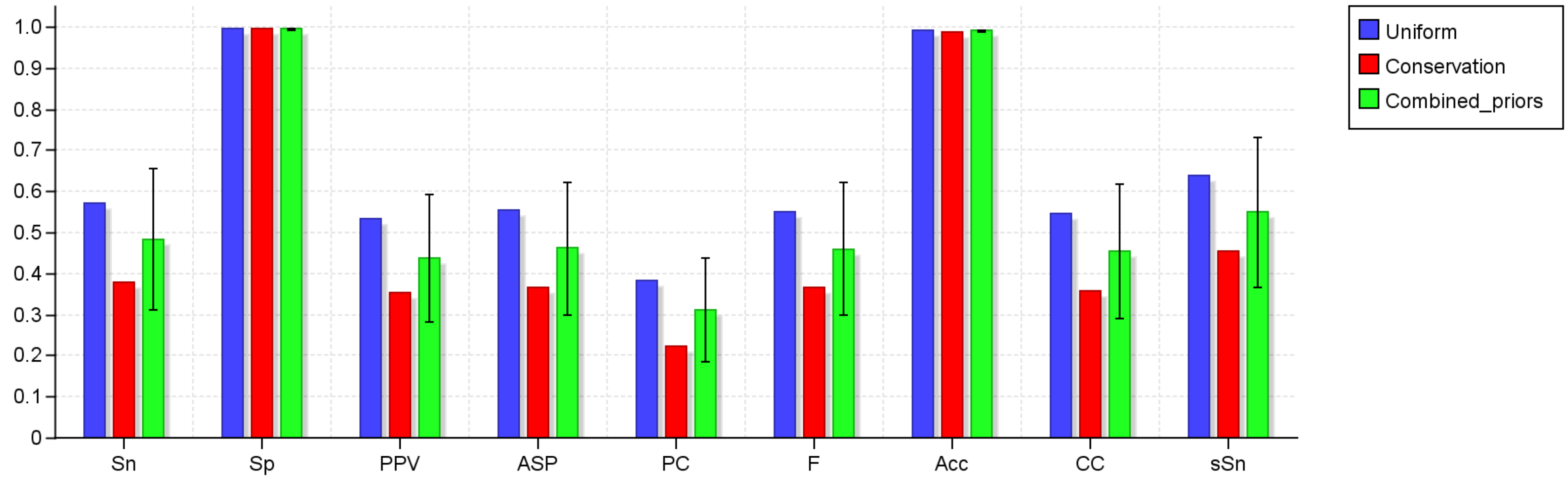

Track	Sn	Sp	PPV	ASP	PC	F	Acc	CC	sSn
**Uniform**	0.571	0.995	0.533	0.552	0.381	0.552	0.991	0.548	0.636
**Conservation**	0.377	0.993	0.352	0.364	0.222	0.364	0.987	0.358	0.455
**Combined_priors**	0.484	0.994	0.437	0.46	0.311	0.459	0.989	0.454	0.55

Figure S1r) Example 1: Dataset “M00983 – MAF”

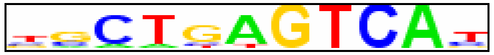

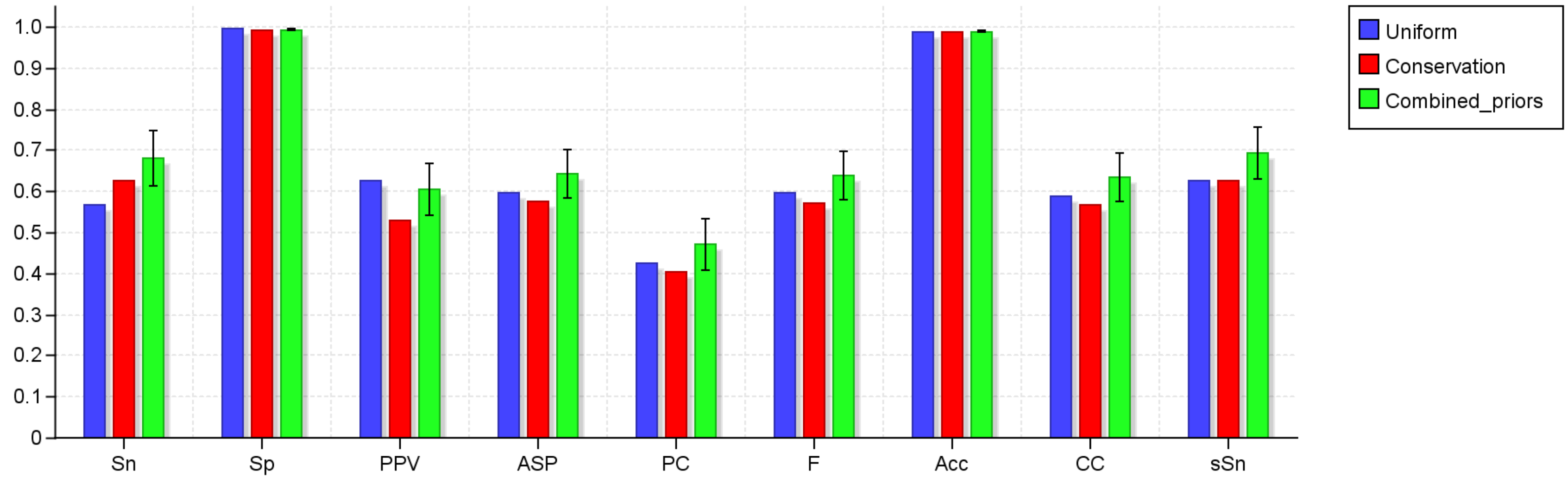

Track	Sn	Sp	PPV	ASP	PC	F	Acc	CC	sSn
**Uniform**	0.568	0.995	0.625	0.597	0.424	0.595	0.988	0.59	0.625
**Conservation**	0.625	0.992	0.529	0.577	0.401	0.573	0.986	0.568	0.625
**Combined_priors**	0.68	0.993	0.604	0.642	0.472	0.638	0.988	0.634	0.694

Figure S1s) Example 1: Dataset “M01007 – SRF” (serum response factor)

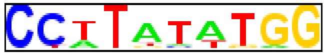

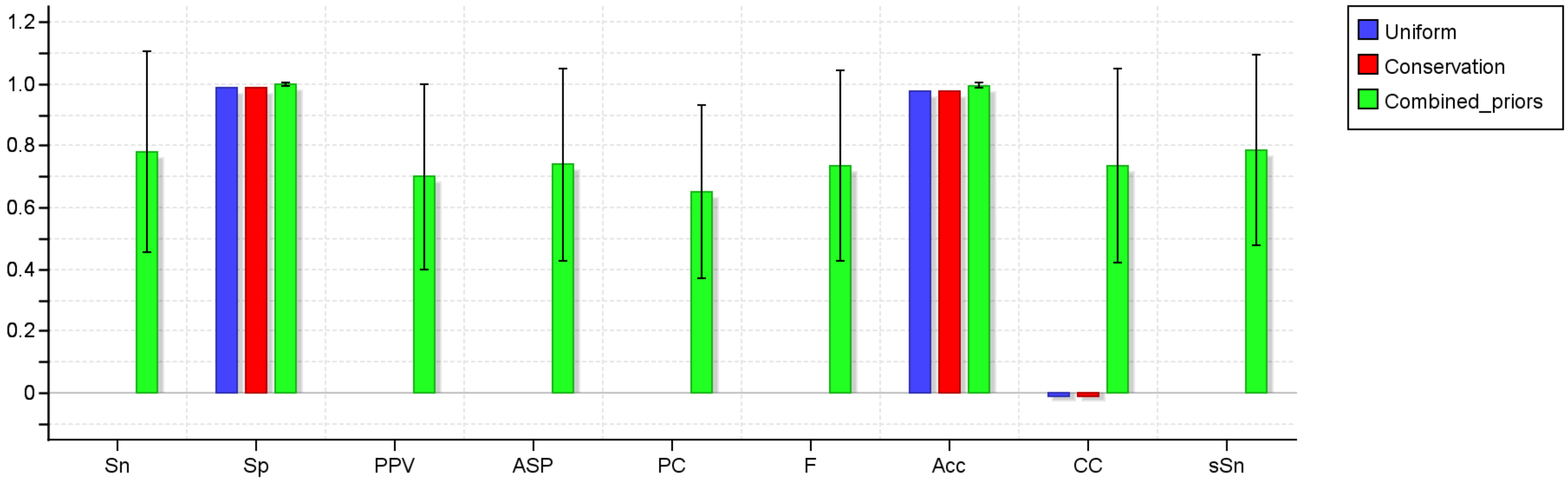

Track	Sn	Sp	PPV	ASP	PC	F	Acc	CC	sSn
**Uniform**	0	0.985	0	0	0	0	0.976	-0.012	0
**Conservation**	0	0.984	0	0	0	0	0.975	-0.012	0
**Combined_priors**	0.779	0.996	0.698	0.739	0.651	0.736	0.994	0.734	0.785

Figure S1t) Example 1: Dataset “M01011 – HNF-1”

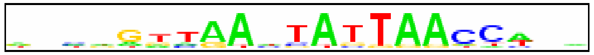

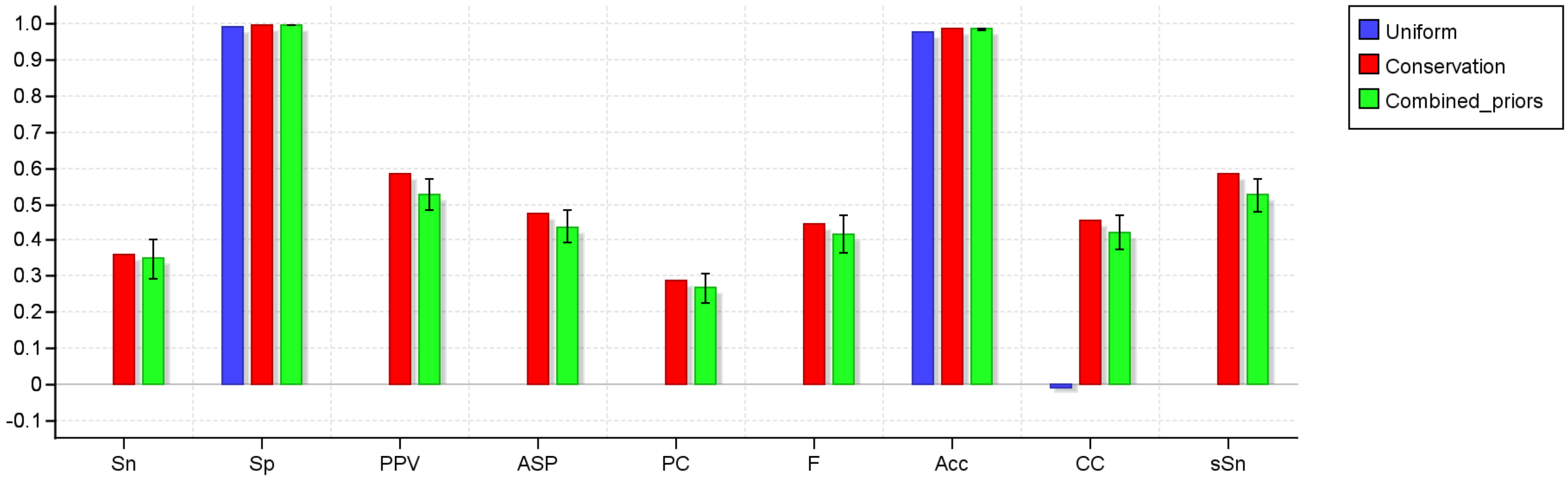

Track	Sn	Sp	PPV	ASP	PC	F	Acc	CC	sSn
**Uniform**	0	0.992	0	0	0	0	0.978	-0.011	0
**Conservation**	0.361	0.996	0.583	0.472	0.287	0.446	0.987	0.453	0.583
**Combined_priors**	0.347	0.995	0.528	0.438	0.265	0.418	0.986	0.421	0.525

Figure S1u) Example 1: Dataset “M01035 – YY1”

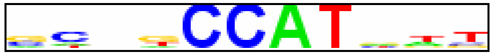

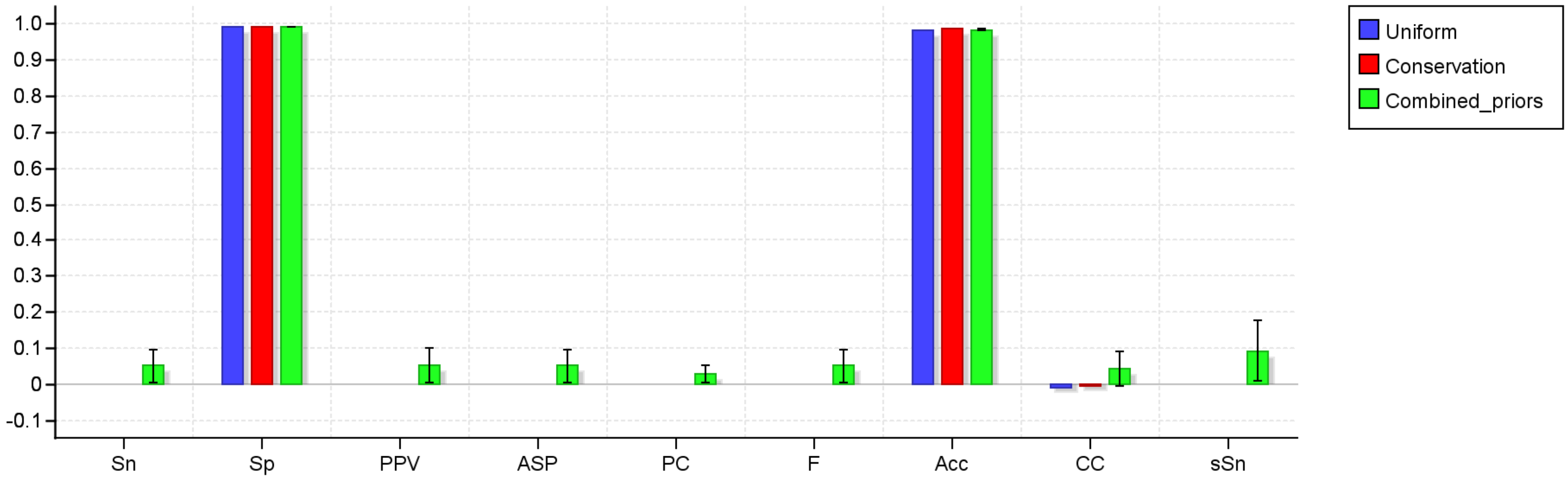

Track	Sn	Sp	PPV	ASP	PC	F	Acc	CC	sSn
**Uniform**	0	0.992	0	0	0	0	0.983	-0.008	0
**Conservation**	0	0.994	0	0	0	0	0.985	-0.007	0
**Combined_priors**	0.05	0.992	0.051	0.051	0.027	0.051	0.984	0.043	0.092

Figure S1v) Example 1: Dataset “M01036 – COUPTF”

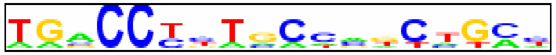

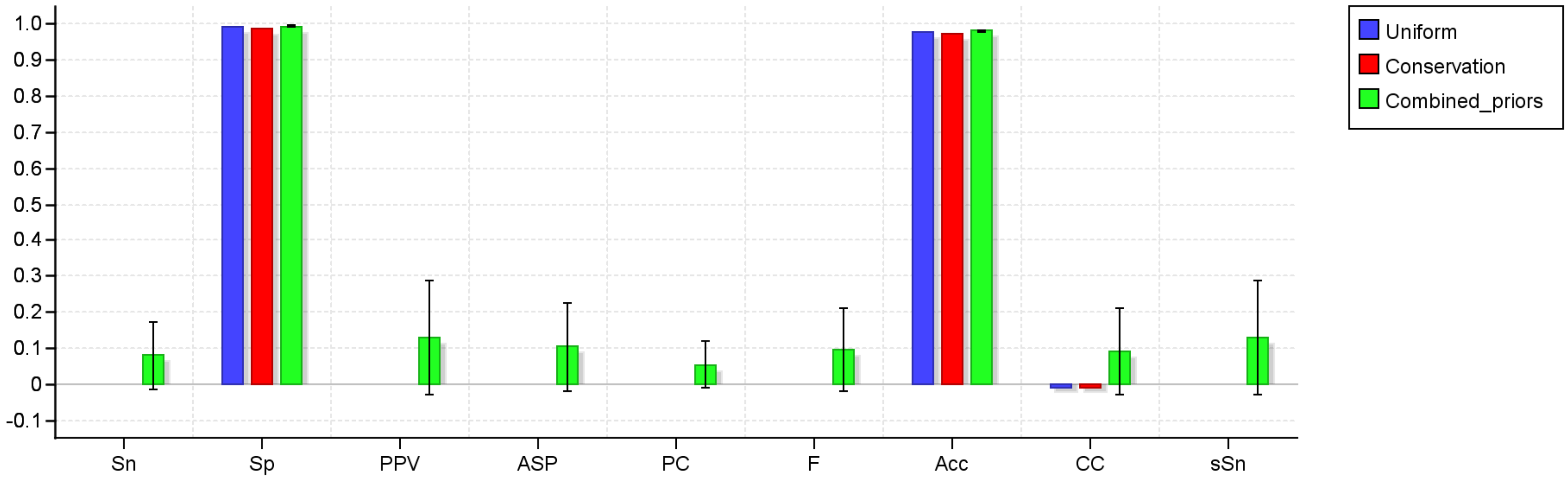

Track	Sn	Sp	PPV	ASP	PC	F	Acc	CC	sSn
**Uniform**	0	0.992	0	0	0	0	0.979	-0.01	0
**Conservation**	0	0.988	0	0	0	0	0.975	-0.012	0
**Combined_priors**	0.078	0.993	0.127	0.103	0.054	0.095	0.981	0.089	0.13

Figure S1w) Example 1: Dataset “M01067 – Gfi1”

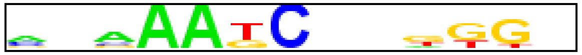

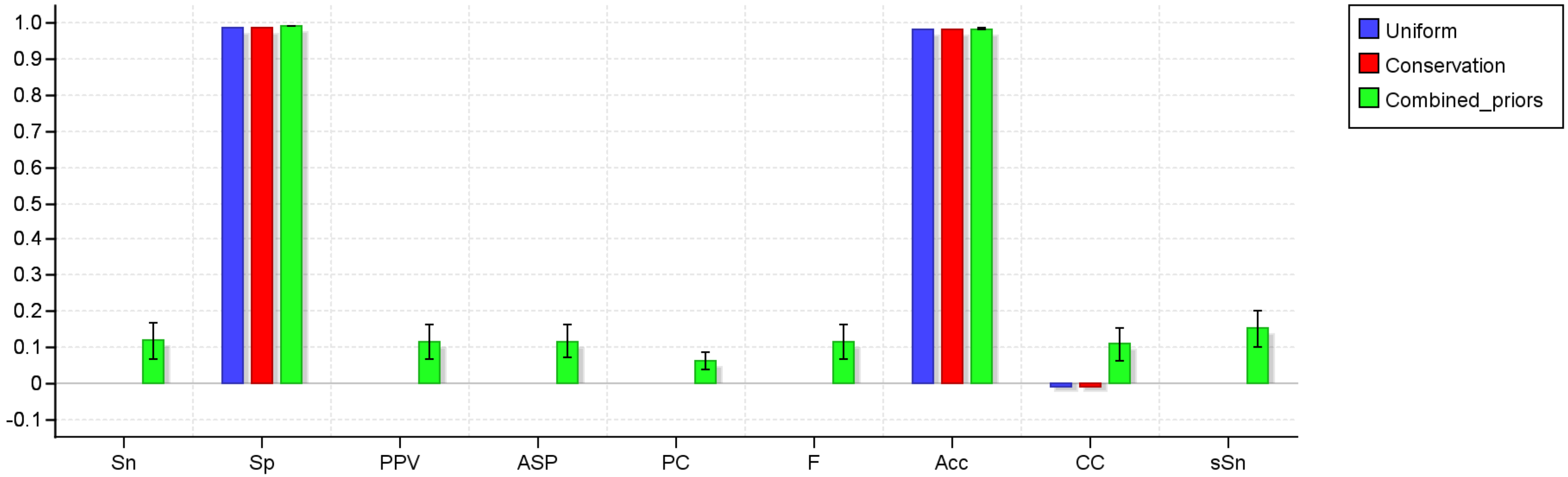

Track	Sn	Sp	PPV	ASP	PC	F	Acc	CC	sSn
**Uniform**	0	0.99	0	0	0	0	0.981	-0.01	0
**Conservation**	0	0.99	0	0	0	0	0.981	-0.01	0
**Combined_priors**	0.117	0.992	0.114	0.116	0.062	0.115	0.985	0.108	0.15

Example 2: Module discovery

The benchmark datasets used for the second example case were based on the composite motif benchmark suite described in Klepper *et al*. [4]. Sequences of viral and bacterial origin in the datasets were discarded and the remaining sequences were mapped to their respective genomes (hg18, mm9, rn4, GalGal3, bosTau4 and oryCun2) with BLAT to find their genomic coordinates. Sequences that could not be mapped uniquely in a satisfactory way were dropped from the datasets. The positions of all target binding sites and modules were verified manually by scanning with the corresponding PWMs for the constituent single motifs. Sequences where the binding sites and modules could not be satisfactory recovered (because of SNPs or indels in the genome builds compared to the original benchmark sequences) were discarded.

We ran the “SimpleScanner” motif scanning method with an 80% match threshold to predict binding sites in all the sequences for 1363 motifs from TRANSFAC Professional. The conservation tracks used to filter candidate sites were based on “phastCons44way” (hg18), “phastCons30way” (mm9), “phastCons9way” (rn4), “phastCons7way” (GalGal3) and “phastCons5way” (bosTau4). We had no conservation track available for rabbit (oryCun2), so for the few sequences originating from this genome the conservation track was just set to 0 everywhere.

For the candidate datasets that were filtered by both conservation and interaction, we applied the interactions filtering before conservation filtering so that binding sites for interaction partners did not have to be conserved.

ModuleSearcher was run with the “genetic algorithm” search heuristic using mostly default parameter settings. For the 10 datasets with modules consisting of pairs of binding sites, we set the module size parameter to “2”, the maximum length parameter to “200bp” and specified that incomplete modules should be penalized. For the more heterogeneous liver and muscle datasets, incomplete modules were not penalized and the module size was set to 4 for the liver dataset (with max length=200bp) and to 5 for the muscle dataset (max length=300bp).

Figure S2a) Example 2: Dataset “AP1 – Ets”

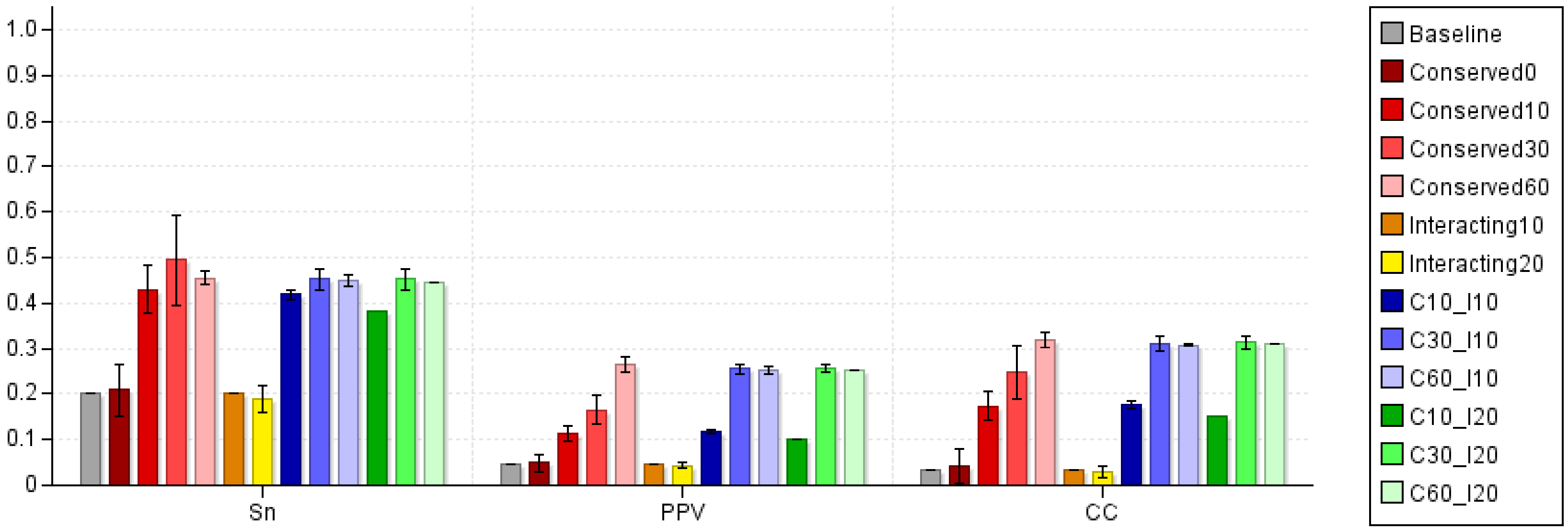

Track	Sn	PPV	CC
**Baseline**	0.2	0.046	0.035
**Conserved0**	0.209	0.049	0.04
**Conserved10**	0.43	0.113	0.174
**Conserved30**	0.494	0.165	0.248
**Conserved60**	0.454	0.264	0.32
**Interacting10**	0.2	0.046	0.035
**Interacting20**	0.19	0.044	0.031

Track	Sn	PPV	CC
**C10_I10**	0.418	0.117	0.177
**C30_I10**	0.452	0.254	0.312
**C60_I10**	0.449	0.252	0.309
**C10_I20**	0.381	0.103	0.151
**C30_I20**	0.453	0.256	0.313
**C60_I20**	0.445	0.254	0.309

Figure S2b) Example 2: Dataset "AP1 – NFAT"

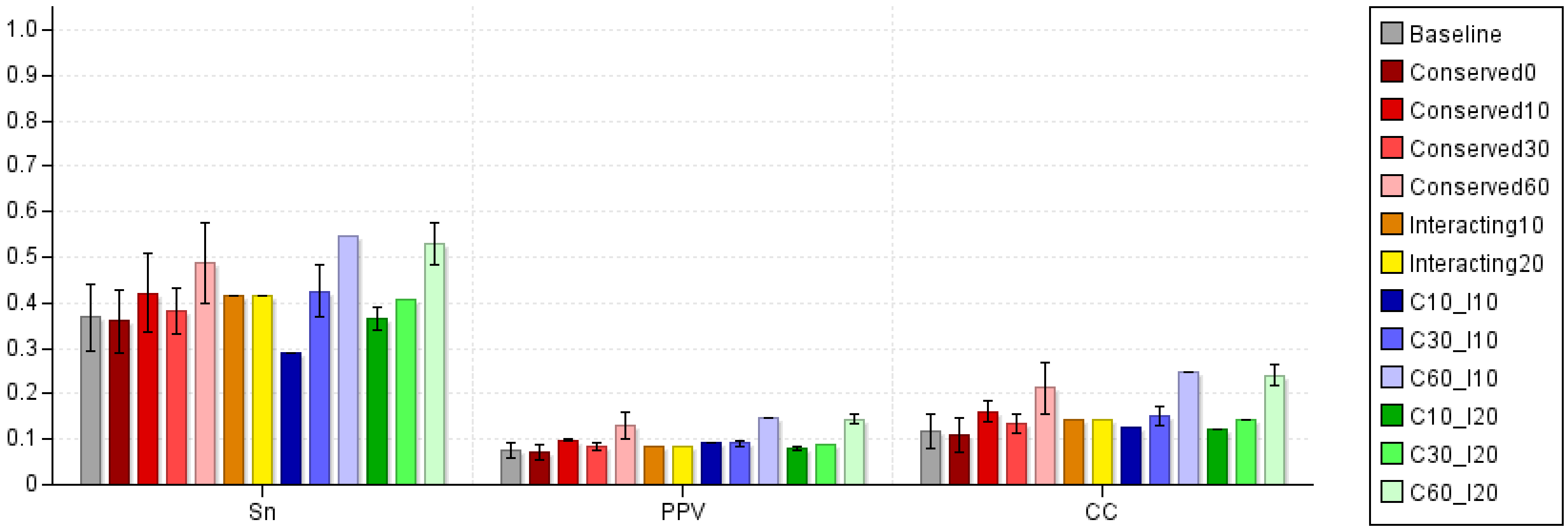

Track	Sn	PPV	CC
**Baseline**	0.369	0.074	0.117
**Conserved0**	0.359	0.072	0.111
**Conserved10**	0.421	0.098	0.161
**Conserved30**	0.382	0.085	0.135
**Conserved60**	0.488	0.129	0.213
**Interacting10**	0.417	0.085	0.141
**Interacting20**	0.417	0.085	0.141

Track	Sn	PPV	CC
**C10_I10**	0.289	0.093	0.125
**C30_I10**	0.424	0.091	0.151
**C60_I10**	0.544	0.148	0.249
**C10_I20**	0.364	0.079	0.123
**C30_I20**	0.406	0.089	0.144
**C60_I20**	0.529	0.144	0.241

Figure S2c) Example 2: Dataset “AP1 – NFkB”

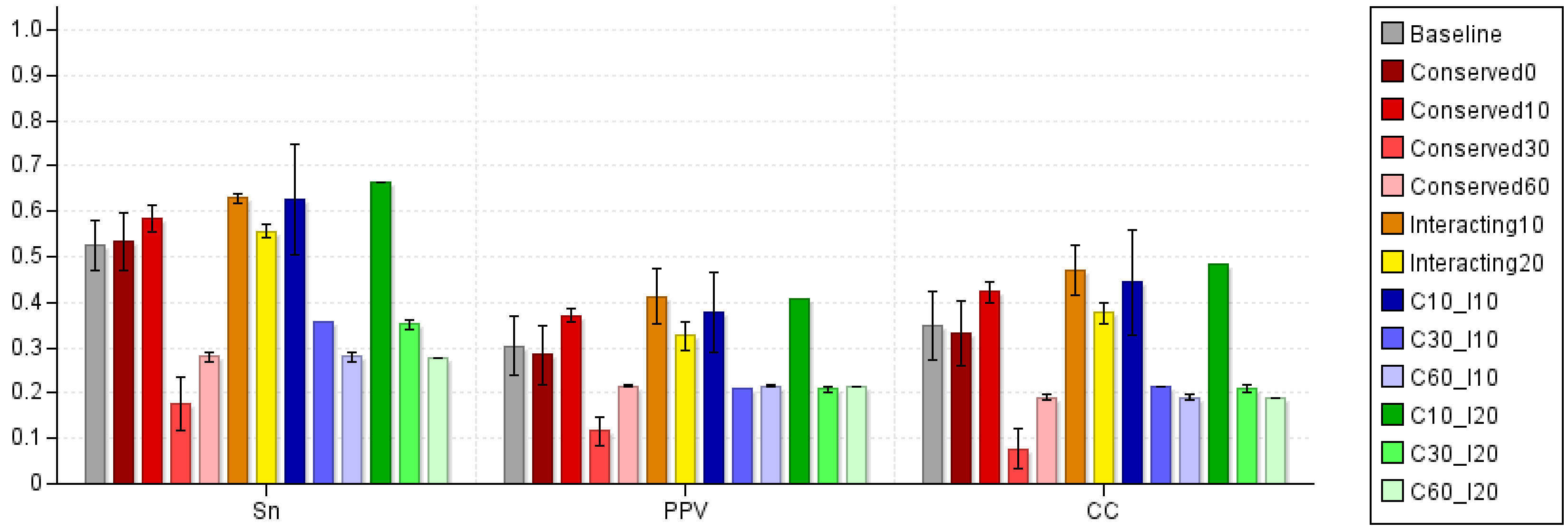

Track	Sn	PPV	CC
**Baseline**	0.524	0.304	0.348
**Conserved0**	0.532	0.284	0.333
**Conserved10**	0.584	0.371	0.424
**Conserved30**	0.177	0.117	0.077
**Conserved60**	0.279	0.215	0.191
**Interacting10**	0.628	0.413	0.469
**Interacting20**	0.556	0.326	0.377

Track	Sn	PPV	CC
**C10_I10**	0.624	0.378	0.443
**C30_I10**	0.356	0.211	0.214
**C60_I10**	0.279	0.215	0.191
**C10_I20**	0.665	0.407	0.482
**C30_I20**	0.352	0.209	0.211
**C60_I20**	0.276	0.214	0.189

Figure S2d) Example 2: Dataset “C/EBP – NFkB”

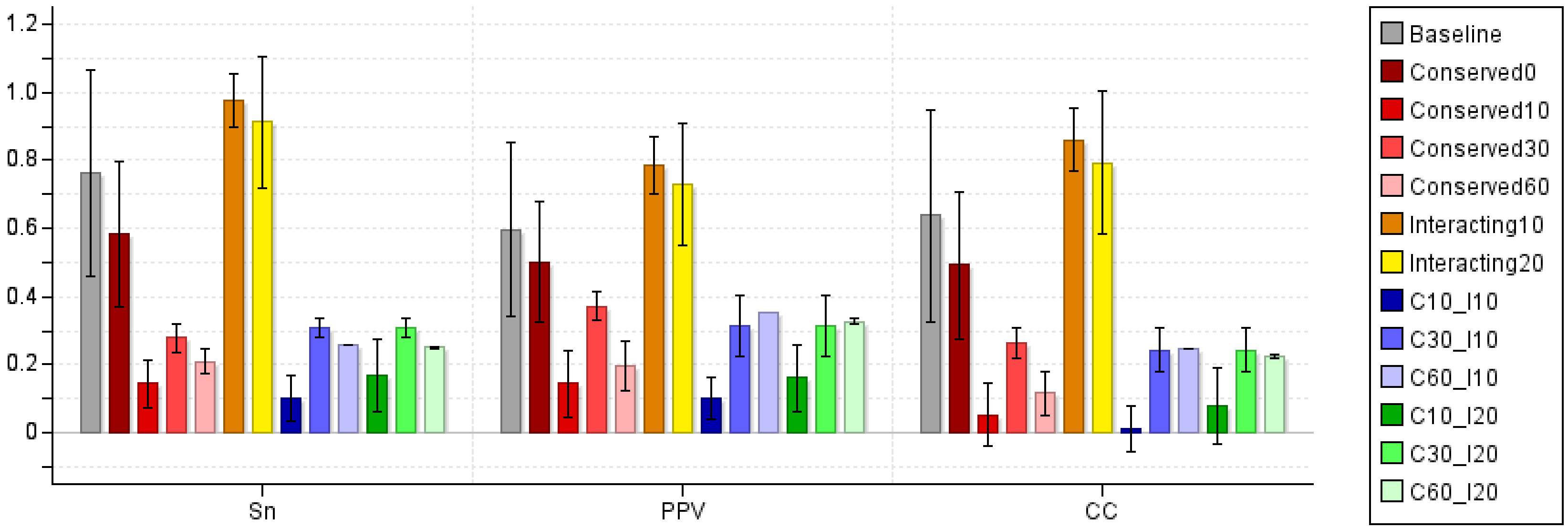

Track	Sn	PPV	CC
**Baseline**	0.763	0.597	0.637
**Conserved0**	0.584	0.502	0.492
**Conserved10**	0.144	0.145	0.053
**Conserved30**	0.279	0.371	0.264
**Conserved60**	0.211	0.196	0.116
**Interacting10**	0.974	0.785	0.861
**Interacting20**	0.911	0.729	0.793

Track	Sn	PPV	CC
**C10_I10**	0.103	0.103	0.013
**C30_I10**	0.31	0.317	0.242
**C60_I10**	0.259	0.357	0.246
**C10_I20**	0.169	0.162	0.081
**C30_I20**	0.31	0.317	0.242
**C60_I20**	0.252	0.328	0.226

Figure S2e) Example 2: Dataset “E-box –Ets”

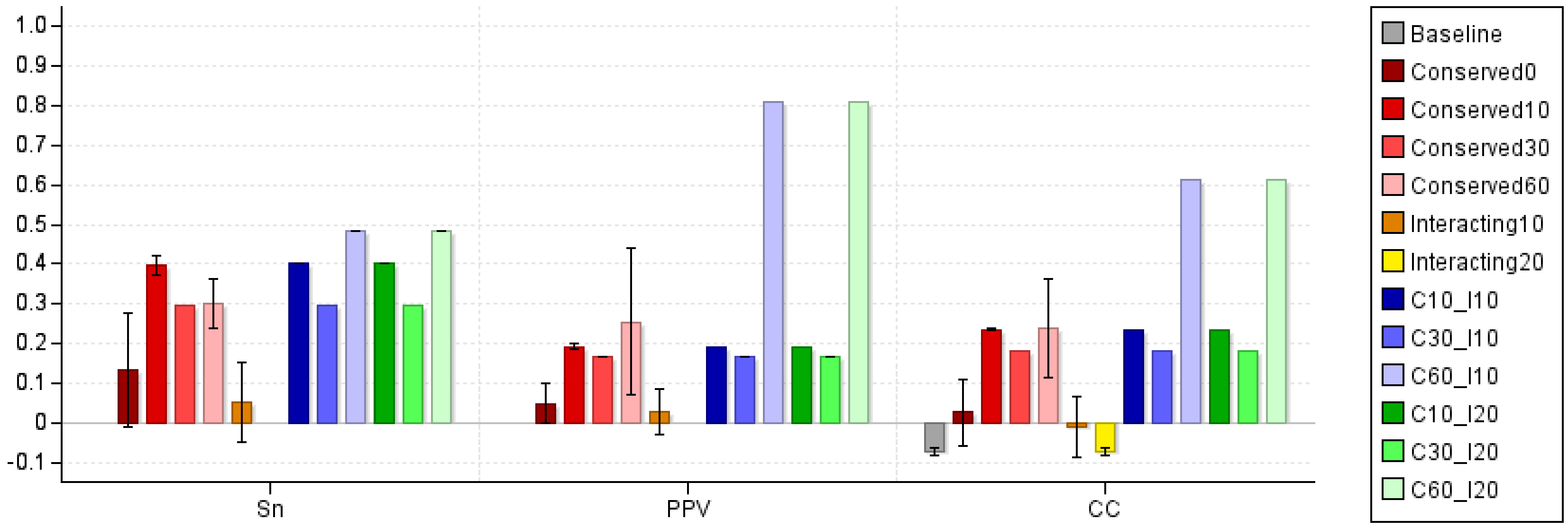

Track	Sn	PPV	CC
**Baseline**	0	0	-0.073
**Conserved0**	0.133	0.049	0.027
**Conserved10**	0.396	0.193	0.235
**Conserved30**	0.298	0.165	0.18
**Conserved60**	0.301	0.255	0.238
**Interacting10**	0.051	0.028	-0.01
**Interacting20**	0	0	-0.075

Track	Sn	PPV	CC
**C10_I10**	0.404	0.191	0.236
**C30_I10**	0.298	0.165	0.18
**C60_I10**	0.482	0.809	0.614
**C10_I20**	0.404	0.191	0.236
**C30_I20**	0.298	0.165	0.18
**C60_I20**	0.482	0.809	0.614

Figure S2f) Example 2: Dataset “Ets –AML”

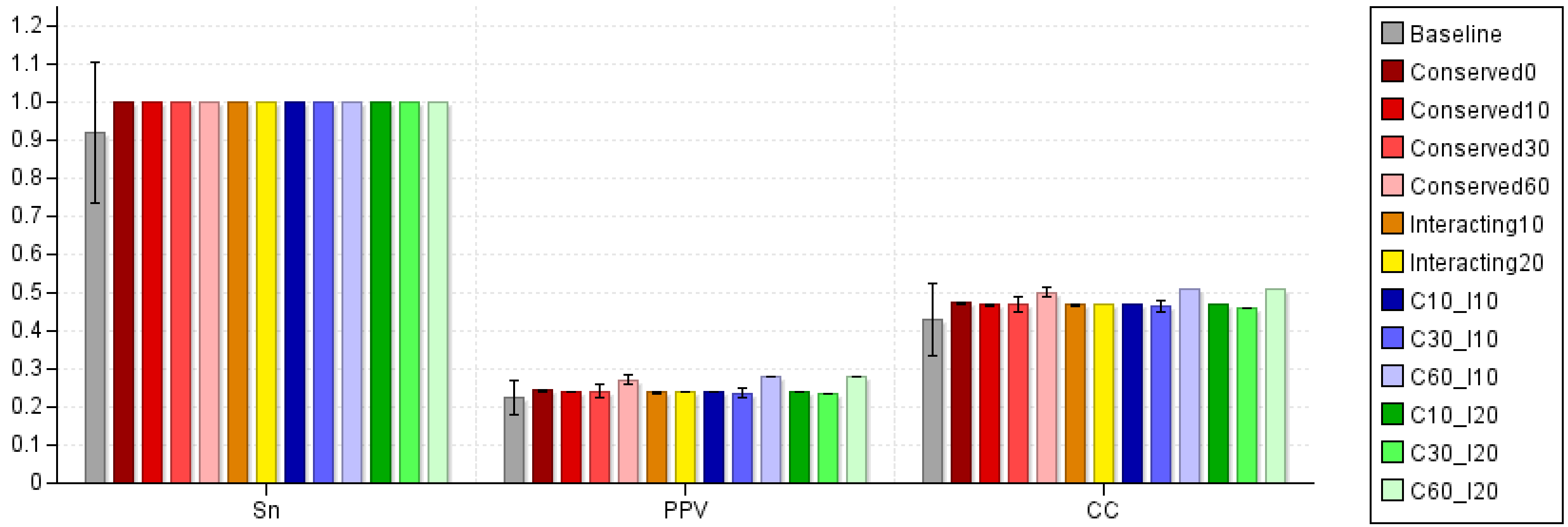

Track	Sn	PPV	CC
**Baseline**	0.921	0.223	0.429
**Conserved0**	1	0.244	0.473
**Conserved10**	1	0.239	0.468
**Conserved30**	1	0.242	0.471
**Conserved60**	1	0.271	0.502
**Interacting10**	1	0.239	0.468
**Interacting20**	1	0.239	0.468

Track	Sn	PPV	CC
**C10_I10**	1	0.239	0.468
**C30_I10**	1	0.237	0.466
**C60_I10**	1	0.278	0.509
**C10_I20**	1	0.239	0.468
**C30_I20**	1	0.233	0.461
**C60_I20**	1	0.278	0.509

Figure S2g) Example 2: Dataset "IRF – NFkB"

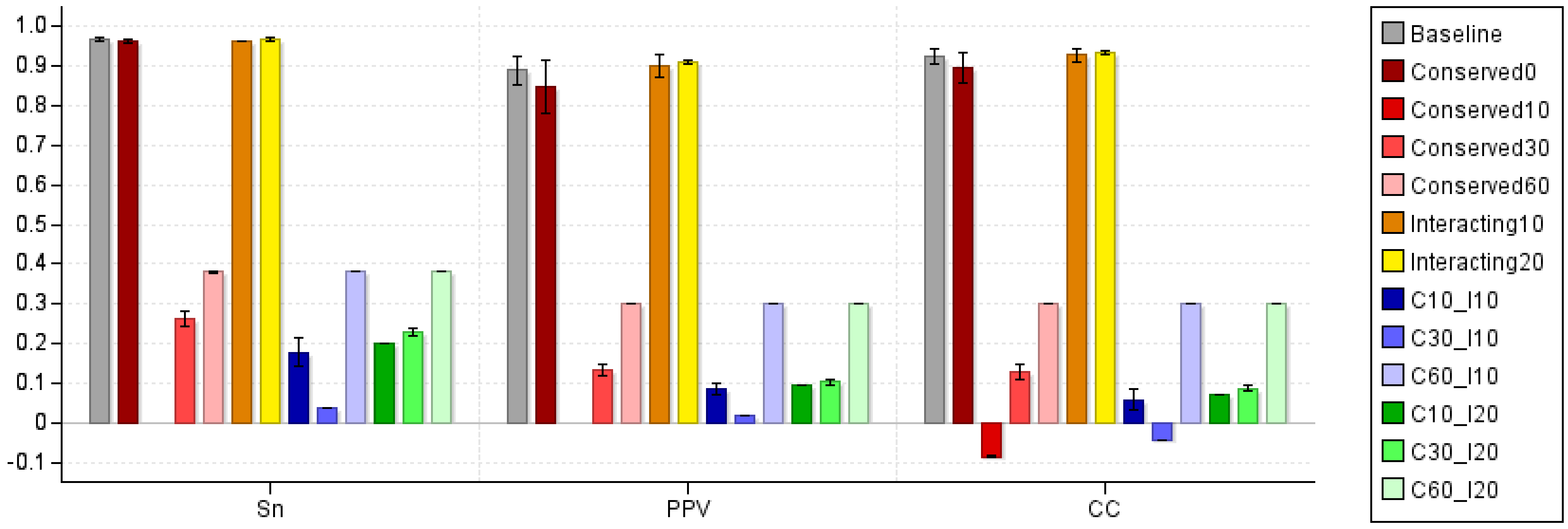

Track	Sn	PPV	CC
**Baseline**	0.967	0.891	0.924
**Conserved0**	0.963	0.847	0.897
**Conserved10**	0	0	-0.086
**Conserved30**	0.262	0.132	0.127
**Conserved60**	0.383	0.303	0.303
**Interacting10**	0.965	0.899	0.928
**Interacting20**	0.967	0.91	0.935

Track	Sn	PPV	CC
**C10_I10**	0.178	0.086	0.058
**C30_I10**	0.039	0.02	-0.042
**C60_I10**	0.384	0.303	0.303
**C10_I20**	0.202	0.095	0.073
**C30_I20**	0.229	0.102	0.087
**C60_I20**	0.384	0.303	0.303

Figure S2h) Example 2: Dataset “NFkB –HMGIY”

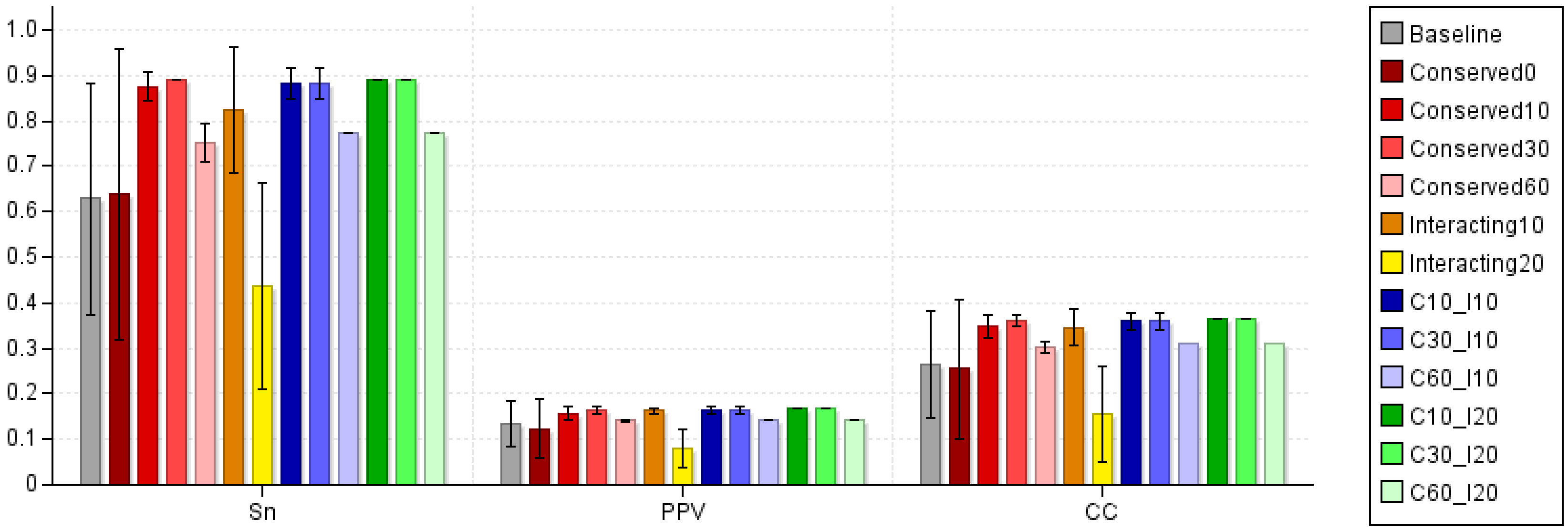

Track	Sn	PPV	CC
**Baseline**	0.629	0.133	0.264
**Conserved0**	0.64	0.124	0.255
**Conserved10**	0.875	0.156	0.349
**Conserved30**	0.892	0.164	0.362
**Conserved60**	0.753	0.141	0.303
**Interacting10**	0.824	0.163	0.345
**Interacting20**	0.437	0.081	0.156

Track	Sn	PPV	CC
**C10_I10**	0.882	0.163	0.36
**C30_I10**	0.882	0.163	0.36
**C60_I10**	0.774	0.143	0.309
**C10_I20**	0.892	0.166	0.365
**C30_I20**	0.892	0.166	0.365
**C60_I20**	0.774	0.143	0.309

Figure S2i) Example 2: Dataset "PU.1 – IRF"

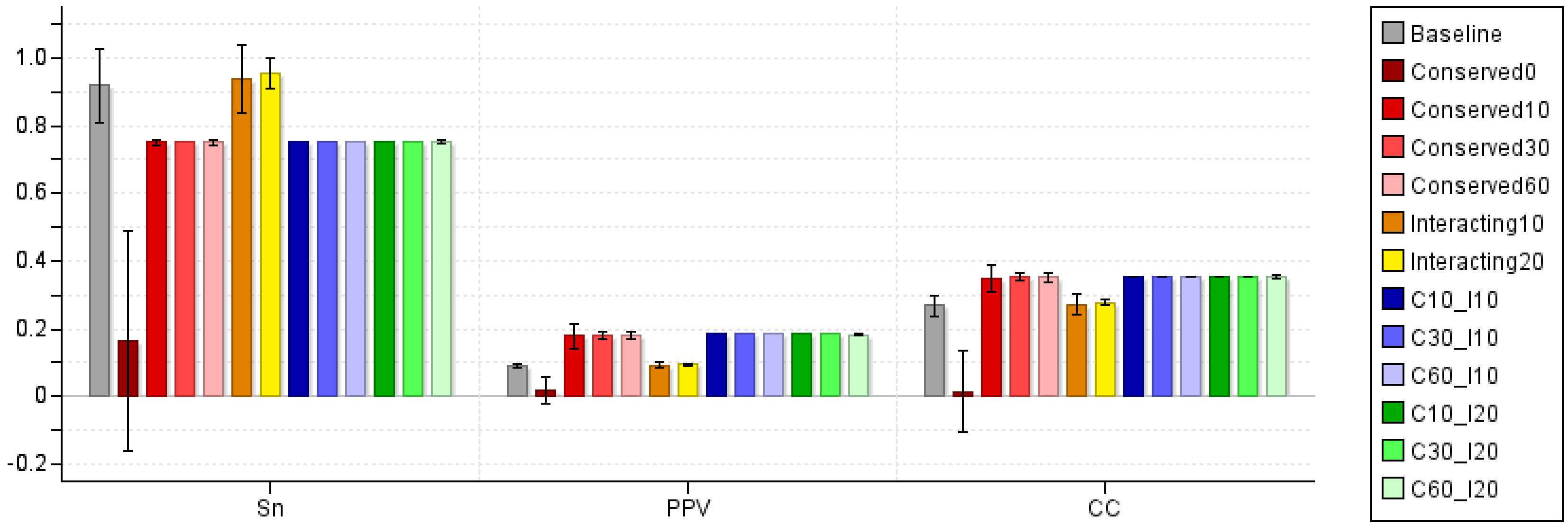

Track	Sn	PPV	CC
**Baseline**	0.918	0.093	0.268
**Conserved0**	0.163	0.019	0.015
**Conserved10**	0.751	0.18	0.349
**Conserved30**	0.754	0.182	0.354
**Conserved60**	0.751	0.181	0.352
**Interacting10**	0.935	0.093	0.272
**Interacting20**	0.954	0.095	0.279

Track	Sn	PPV	CC
**C10_I10**	0.754	0.185	0.358
**C30_I10**	0.754	0.185	0.358
**C60_I10**	0.754	0.185	0.358
**C10_I20**	0.754	0.185	0.358
**C30_I20**	0.754	0.185	0.358
**C60_I20**	0.752	0.184	0.356

Figure S2j) Example 2: Dataset "SP1 – Ets"

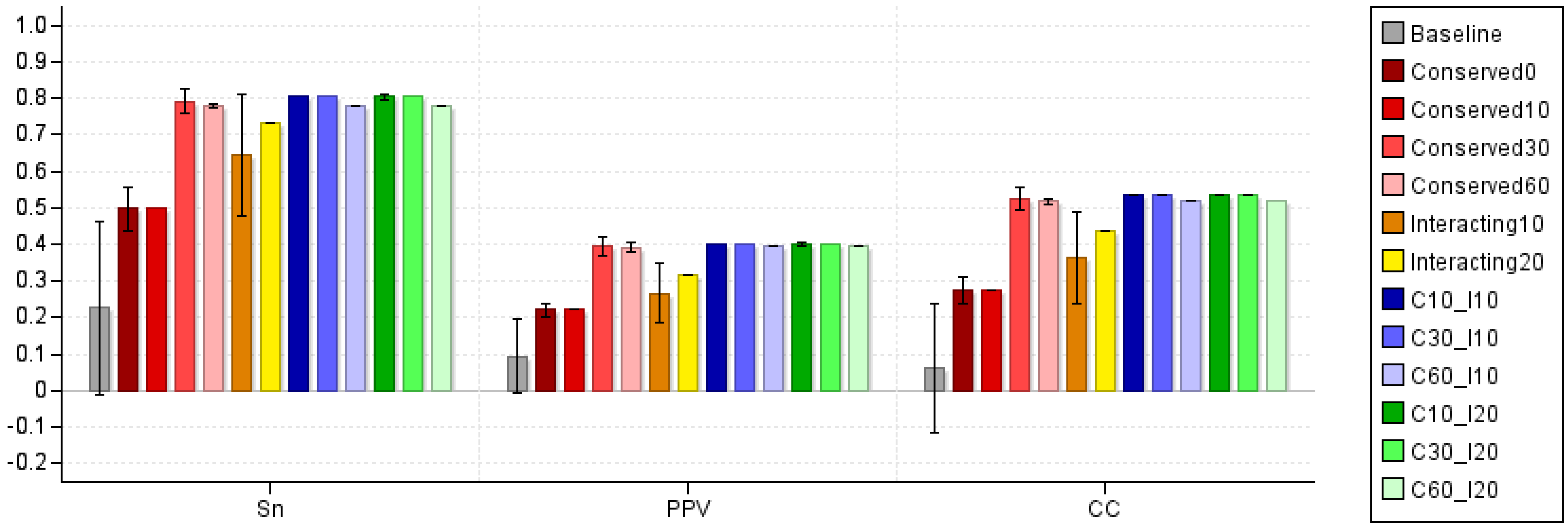

Track	Sn	PPV	CC
**Baseline**	0.227	0.094	0.062
**Conserved0**	0.497	0.223	0.276
**Conserved10**	0.498	0.224	0.277
**Conserved30**	0.792	0.395	0.525
**Conserved60**	0.781	0.391	0.518
**Interacting10**	0.644	0.267	0.363
**Interacting20**	0.734	0.315	0.438

Track	Sn	PPV	CC
**C10_I10**	0.806	0.398	0.534
**C30_I10**	0.806	0.398	0.534
**C60_I10**	0.779	0.395	0.521
**C10_I20**	0.803	0.4	0.534
**C30_I20**	0.806	0.398	0.534
**C60_I20**	0.779	0.395	0.521

Figure S2k) Example 2: Dataset "Liver"

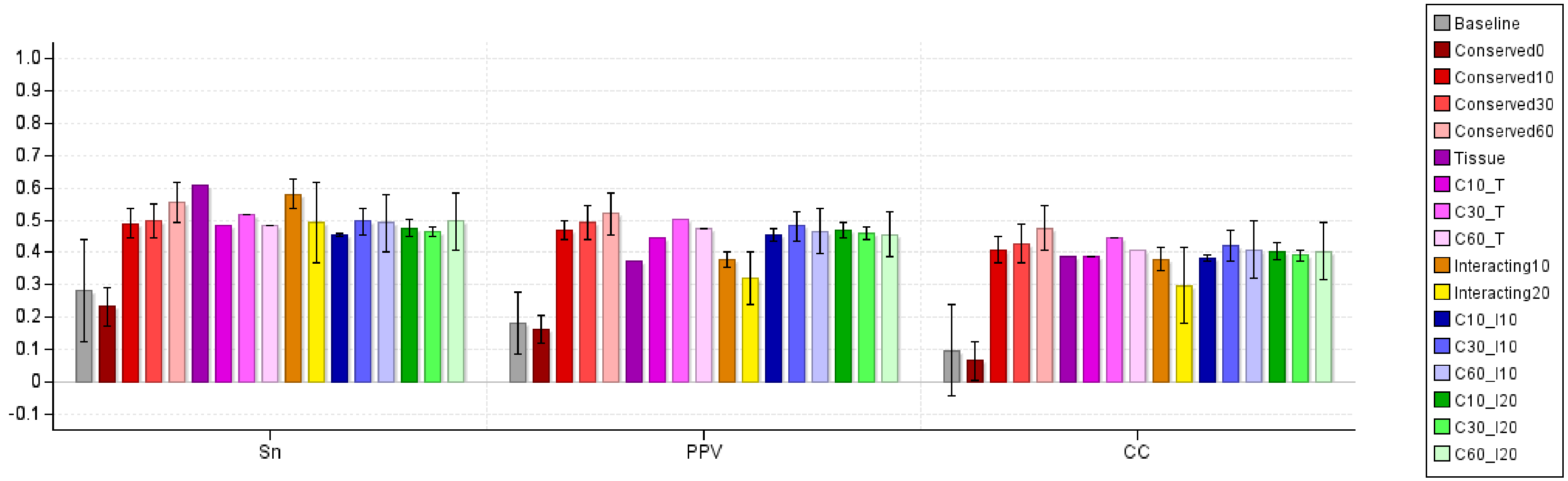

Track	Sn	PPV	CC
**Baseline**	0.28	0.18	0.097
**Conserved0**	0.232	0.163	0.065
**Conserved10**	0.49	0.468	0.409
**Conserved30**	0.498	0.492	0.428
**Conserved60**	0.556	0.522	0.476
**Tissue**	0.609	0.375	0.39
**C10_T**	0.484	0.444	0.39
**C30_T**	0.516	0.503	0.444
**C60_T**	0.485	0.472	0.409

Track	Sn	PPV	CC
**Interacting10**	0.581	0.377	0.38
**Interacting20**	0.493	0.321	0.298
**C10_I10**	0.455	0.455	0.383
**C30_I10**	0.498	0.483	0.422
**C60_I10**	0.492	0.466	0.408
**C10_I20**	0.476	0.469	0.403
**C30_I20**	0.463	0.46	0.391
**C60_I20**	0.496	0.456	0.404

Figure S2l) Example 2: Dataset “Muscle”

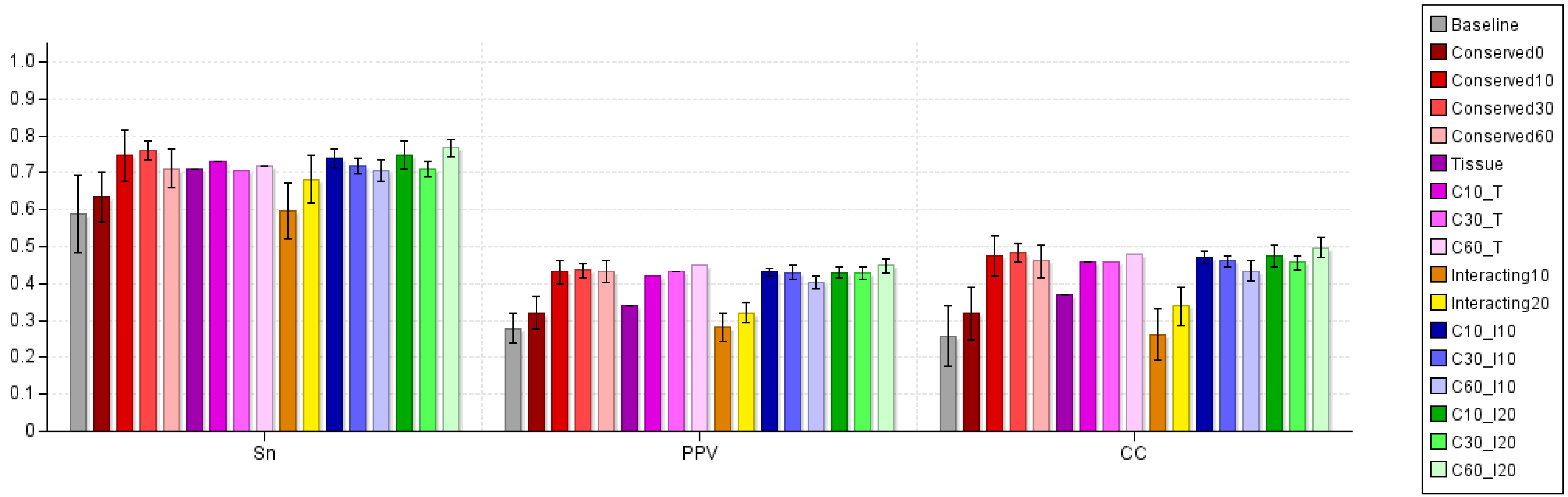

Track	Sn	PPV	CC
**Baseline**	0.588	0.278	0.258
**Conserved0**	0.633	0.321	0.32
**Conserved10**	0.746	0.431	0.474
**Conserved30**	0.759	0.436	0.483
**Conserved60**	0.711	0.432	0.46
**Tissue**	0.711	0.338	0.368
**C10_T**	0.733	0.421	0.459
**C30_T**	0.706	0.434	0.46
**C60_T**	0.719	0.451	0.48

Track	Sn	PPV	CC
**Interacting10**	0.596	0.28	0.262
**Interacting20**	0.682	0.321	0.339
**C10_I10**	0.739	0.431	0.47
**C30_I10**	0.717	0.43	0.46
**C60_I10**	0.706	0.405	0.433
**C10_I20**	0.748	0.43	0.474
**C30_I20**	0.71	0.428	0.456
**C60_I20**	0.767	0.448	0.497

References

1. Sandve GK, Abul O, Walseng V, Drablos F: **Improved benchmarks for computational motif discovery.** *BMC Bioinformatics* 2007, **8:**193.
2. Kent WJ: **BLAT--the BLAST-like alignment tool.** *Genome Res* 2002, **12:**656-664.
3. Dreszer TR, Karolchik D, Zweig AS, Hinrichs AS, Raney BJ, Kuhn RM, Meyer LR, Wong M, Sloan CA, Rosenbloom KR, et al: **The UCSC Genome Browser database: extensions and updates 2011.** *Nucleic Acids Res* 2012, **40:**D918-923.
4. Klepper K, Sandve GK, Abul O, Johansen J, Drablos F: **Assessment of composite motif discovery methods.** *BMC Bioinformatics* 2008, **9:**123.
